# Exploring the Impact of *Onobrychis cornuta* and *Veratrum lobelianum* Extracts on *C. elegans*: Implications for MAPK Modulation, Germline Development, and Antitumor Properties

**DOI:** 10.3390/nu16010008

**Published:** 2023-12-19

**Authors:** Qinghao Meng, Nishit Pathak, Xiaojing Ren, Robert P. Borris, Hyun-Min Kim

**Affiliations:** 1 School of Pharmaceutical Science and Technology, Tianjin University, Tianjin 300072, China; mengqh@tju.edu.cn (Q.M.); npathak89@gmail.com (N.P.); borrisr@gmail.com (R.P.B.); 2 Division of Natural and Applied Sciences, Duke Kunshan University, Kunshan 215316, China

**Keywords:** *O. cornuta*, V. lobelianum, DNA repair, meiosis, linoleic acid, germline

## Abstract

In an era of increasing interest in the potential health benefits of medicinal foods, the need to assess their safety and potential toxicity remains a critical concern. While these natural remedies have garnered substantial attention for their therapeutic potential, a comprehensive understanding of their effects on living organisms is essential. We examined 316 herbal extracts to determine their potential nematocidal attributes in *Caenorhabditis elegans*. Approximately 16% of these extracts exhibited the capacity to induce diminished survival rates and larval arrest, establishing a correlation between larval arrest and overall worm viability. Certain extracts led to an unexpected increase in male nematodes, accompanied by a discernible reduction in DAPI-stained bivalent structures and perturbed meiotic advancement, thereby disrupting the conventional developmental processes. Notably, *Onobrychis cornuta* and *Veratrum lobelianum* extracts activated a DNA damage checkpoint response via the ATM/ATR and CHK-1 pathways, thus hindering germline development. Our LC–MS analysis revealed jervine in *V. lobelianum* and nine antitumor compounds in *O. cornuta*. Interestingly, linoleic acid replicated phenotypes induced by *O. cornuta* exposure, including an increased level of pCHK-1 foci, apoptosis, and the MAPK pathway. Mutants in the MAPK pathway mitigated the decline in worm survival, underscoring its importance in promoting worm viability. This study reveals complex interactions between herbal extracts and *C. elegans* processes, shedding light on potential antitumor effects and mechanisms. The findings provide insights into the complex landscape of herbal medicine’s impact on a model organism, offering implications for broader applications.

## 1. Introduction

Medicinal foods are a rapidly growing market with an expected annual size of USD 2.1 billion, projected to expand by 5.2% from 2022 to 2030 [1]. Despite their effectiveness, it is important to note that many herbs can be toxic and lead to side effects. Moreover, numerous herbs have not undergone thorough analytical examination and have not received approval from the Food and Drug Administration (FDA) for use as medicine. Consequently, understanding how herbs function and the potential risks associated with their usage becomes crucial.

To assess the potential toxicity of herbal drugs, various model systems have been employed. The utilization of *C. elegans* offers several advantages due to its simplistic system that allows the comprehension of complex processes [2,3,4]. With high genetic homology (60–80%) to humans; conserved major biological pathways; and the availability of numerous genetic tools, such as transgenic models, gene knockouts, and RNAi depletions, *C. elegans* serves as a valuable multicellular animal model for studying human diseases and aging [5,6]. Moreover, its substantial brood size (~300 progeny), short lifespan, and range of behavioral phenotypes provide additional benefits for drug screening while minimizing maintenance costs [7]. The transparent skin of *C. elegans* and larger germline volume also offer advantages for studying meiotic progression. Due to these attributes, *C. elegans* has contributed to numerous significant discoveries in aging research [8].

In recent studies, *C. elegans* has been utilized to explore the effects of herbal extracts. For instance, rosemary flower-derived phenolic compositions were found to alleviate oxidative stress and extend lifespan [9]. Additionally, *E. ulmoides* and *C. chinensis* extracts enhanced worm survival under heat stress and exposure to pathogens [10]. Ayurvedic herbal extracts displayed neuroprotective and protein aggregation-mitigating effects, making them potentially beneficial for alleviating symptoms of Parkinson’s disease [11].

In this study, we investigated 316 herbal extracts for potential antitumor properties in *C. elegans*. Some (~16%) of the extracts exhibited reduced survival and larval arrest/lethality, indicating that larval arrest plays a role in determining worm viability. Surprisingly, a small but significant portion of herb extracts demonstrated a high incidence of males (HIM), a reduced number of DAPI-stained bodies, and faulty meiotic progression, indicating that certain worms also undergo aberrant meiotic development upon herbal treatment. Interestingly, some herbal extracts triggered a response from the DNA damage checkpoint. When worms were treated with *O. cornuta* and *V. lobelianum*, a DNA damage checkpoint response was activated via the ATM/ATR and CHK-1 pathways and was accompanied by impaired germline development. These findings suggest that both *O. cornuta* and *V. lobelianum* extracts hinder the process of DNA damage repair.

We further examined whether compounds in *O. cornuta* and *V. lobelianum* contribute to their antitumor properties. To delve deeper into the composition of the two herbal extracts, we conducted an analysis, using LC–MS. Consistent with a previous report, jervine was identified in the *V. lobelianum* extract. Surprisingly, the *O. cornuta* extract revealed the presence of nine known antitumor compounds. The *O. cornuta* extract and the five major compounds found in *O. cornuta* exhibit upregulation of the MAPK kinase components MEK-2 and SOS-1. Moreover, linoleic acid further mimicked most of the phenotypes exhibited in *O. cornuta*, including increased levels of pCHK-1 and apoptosis. Additionally, mutants defective in the MAPK pathway suppressed the phenotypes observed in *O. cornuta* exposure, suggesting that the MAPK pathway is responsible for survivability and DNA damage checkpoint apoptosis.

## 2. Results

### 2.1. Herbal Extracts Unveil Nematocidal Potency

**Figure 1:**
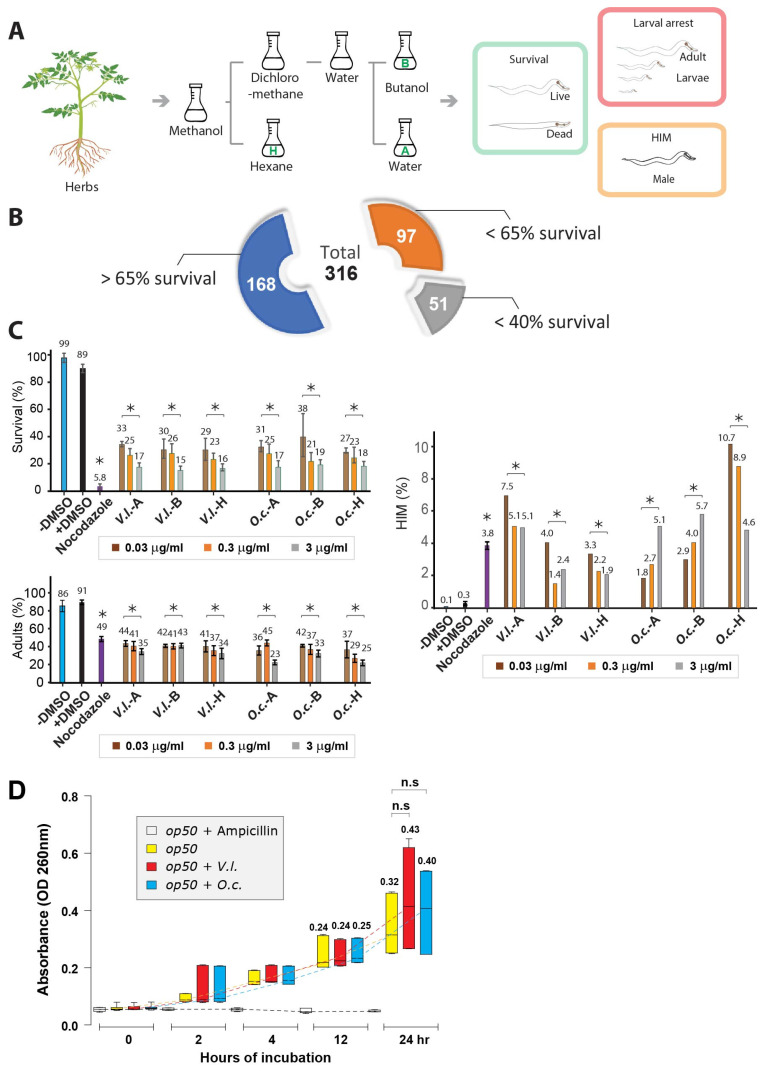
Herb extracts have an inhibitory effect on worm survival and development but do not affect bacterial growth. (**A**) Experimental workflow depicting the process of extracting compounds from herbs. The extracted compounds were then subjected to a phenotypic analysis, using the *C. elegans* model system. A 1 kg sample of the air-dried whole plant of each plant was milled to a coarse powder and extracted with methanol (3 × 6 L). The pooled methanol extract was concentrated in vacuo to afford a tarry residue. The methanol extract was dissolved in 90% (aqueous) methanol and extracted with n-hexane. The residual hydroalcoholic phase was freed of the solvent in vacuo, suspended in water, and then sequentially extracted with dichloromethane and n-butanol (n-BuOH) to afford a gross separation into hexane-, dichloromethane-, butanol-, and water-soluble fractions. Three solvents, i.e., hexane, butanol, and water, were designated -H, -B, and -A, respectively. The herbal extracts used in the screening process are listed in Supplementary Table S1. (**B**) The impact of plant extracts on *C. elegans* survival and development overview. A total of 316 (168 + 97 + 51) extracts were tested, revealing significant nematocidal effects. Survival (%), adult (%), and HIM (%) of *C. elegans* were monitored after a 24 h treatment of herb extract and monitored for 48 h. (**C**) Dose-dependent effects of herb extracts on survival, adult formation, and HIM phenotype. Investigation into the dose-dependent relationship between herbal extract concentrations and *C. elegans* phenotypes. (Top, middle) Survival rates and adult formation were inversely correlated with increasing herbal extract doses. Dose–response trends were consistent across extracts from different solvents (-A, -B, and -H). (Bottom) Induction of the HIM phenotype in response to herb extracts. *V.l.* and *O.c.* extracts were treated at a final concentration of 0.03, 0.3, and 3 µg/mL. The *p*-values were determined by the two-tailed Mann–Whitney test. Statistical significance between +DMSO and the samples is indicated by asterisks, using the two-tailed *t*-test. (**D**) Evaluation of herb extracts’ impact on bacterial growth. Analysis over 24 h revealed no significant bacterial growth defect, suggesting that the nematocidal effects were not primarily attributed to compromised bacterial growth. *V.I* and *O.c* were added to a final concentration of 0.03 μg/mL. Asterisks indicate statistically significant.

We utilized 316 different herbs to prepare herbal extracts and evaluated their cytotoxic effects on the nematode *C. elegans* (Figure 1A,B). For herb extraction, we employed the solvents hexane, butanol, and water, denoted as -H, -B, and -A, respectively.

Almost half of the plant extracts (47%, 148 out of 316 extracts) demonstrated potent nematocidal effects following a 24 h treatment at 20 °C, resulting in less than 65% survival (Figure 1B). Moreover, among these extracts, 51 (16%) exhibited even lower survival rates, falling below 40%, in comparison to a control group treated with DMSO. We directed our attention toward two distinctive herbal extracts, *O. cornuta (O.c.*) and *V. lobelianum (V.l.*), both of which exhibited notable phenotypes (Figure 1C). The comparative analysis revealed that both herbal extracts exhibited significantly reduced survivability when compared to the nontreated control group (89 vs. 33 in +DMSO and *V.l.*-A, *p* = 0.003; 89 vs. 27 in +DMSO and *O.c.*-H, *p* = 0.003, as determined by the two-tailed Mann–Whitney test).

Furthermore, worms exposed to these extracts demonstrated either larval arrest and/or lethality (91 vs. 44 in +DMSO and *V.l.*-A, *p* = 0.004; 91 vs. 37 in +DMSO and *O.c.*-H, *p* = 0.004, using the two-tailed Mann–Whitney test). This observation strongly suggests a link between compromised survivability and mitotic growth defects. Notably, both extracts also induced a significant increase in the occurrence of males (HIM phenotype), indicating potential sex chromosome mis-segregation and abnormal meiotic development in the worms (0.3 vs. 7.5 in +DMSO and *V.l.*-A, *p* = 0.005; 0.3 vs. 10.7 in +DMSO and *O.c.*-H, *p* = 0.004, as determined by the two-tailed Mann–Whitney test).

### 2.2. Dose-Dependent Nematocidal Effects of Herbal Extracts: Correlation and Phenotypic Observations

To further validate the nematocidal effects, we investigated whether varying doses of herb extracts correlated with the observed phenotypes of the two herbs. As the dose of the herbal extract increased tenfold, we observed a corresponding decrease in survivability for both herbal extracts, indicating a clear dose-dependent phenomenon (Figure 1C). For instance, in *V.l.*-A, survivability changed from 33 to 25 to 17, and in *O.c.*-H, it changed from 27 to 23 to 18 for concentrations of 0.03, 0.3, and 3 ng/mL, respectively.

Remarkably, this dose dependency was evident across extracts obtained from three solvents (A, B, and H). Similarly, the number of adults also declined with the increasing herb concentrations, indicating that the herb extracts indeed impeded mitotic growth (Figure 1C). For instance, in *V.l.*-A, the number of adults shifted from 44 to 41 to 35, and in *O.c.*-H, it shifted from 37 to 29 to 25 for concentrations of 0.03, 0.3, and 3 ng/mL, respectively.

*C. elegans* reproduce through self-fertilization. Errors during the separation of sex chromosomes in cell division can lead to offspring with abnormal sex chromosome compositions, resulting in an increased incidence of males, known as the HIM phenotype. This HIM phenotype serves as a crucial indicator for studying the impacts of these factors on sex chromosome segregation, chromosomal abnormalities, and reproductive processes [3,12].

While we observed the induction of the HIM phenotype with all six types of herb extracts, intriguingly, we did not discern a distinct dose dependency. This suggests that defective sex chromosomal segregation was not directly correlated with the dosages of the herb extracts (Figure 1C). For example, in *V. lobelianum*-A, the HIM phenotype was observed at 7.5, 5.1, and 5.1, and in *O.c.*-H, it was observed at 10.7, 8.9, and 4.6 for concentrations of 0.03, 0.3, and 3 ng/mL, respectively.

### 2.3. Impact of Herb Extracts on Nematode Survival and Bacterial Growth

The decreased survival of *C. elegans* might arise from poor bacterial growth rather than a direct correlation between worms and herbs. To explore this possibility, we investigated whether herb extracts inhibit bacterial growth. No significant bacterial growth defect was observed in the case of the two herbs over 24 h of incubation, implying that bacterial growth did not play a crucial role in the nematocidal effects (Figure 1D, *p* < 0.1248 in OP50 and OP50 +*V.l.*; *p* < 0.1994 in OP50 and OP50 +*O.c.*).

Our data indicate that both *V. lobelianum* and *O. cornuta* extracts hinder survivability, larval growth, and sex chromosomal segregation. The dosage of herb extracts correlates with decreased survivability and larval arrest.

### 2.4. Herbal Extracts Cause Defective Germline Progression

The nuclei within *C. elegans* are spatially and temporally arranged along germline progression, with actively dividing mitotic nuclei occupying the distal end of the premeiotic tip (PMT). As cells move away from the PMT, they enter meiotic prophase, commencing from the transition zone, where nuclei assume a crescent shape [3,13].

**Figure 2:**
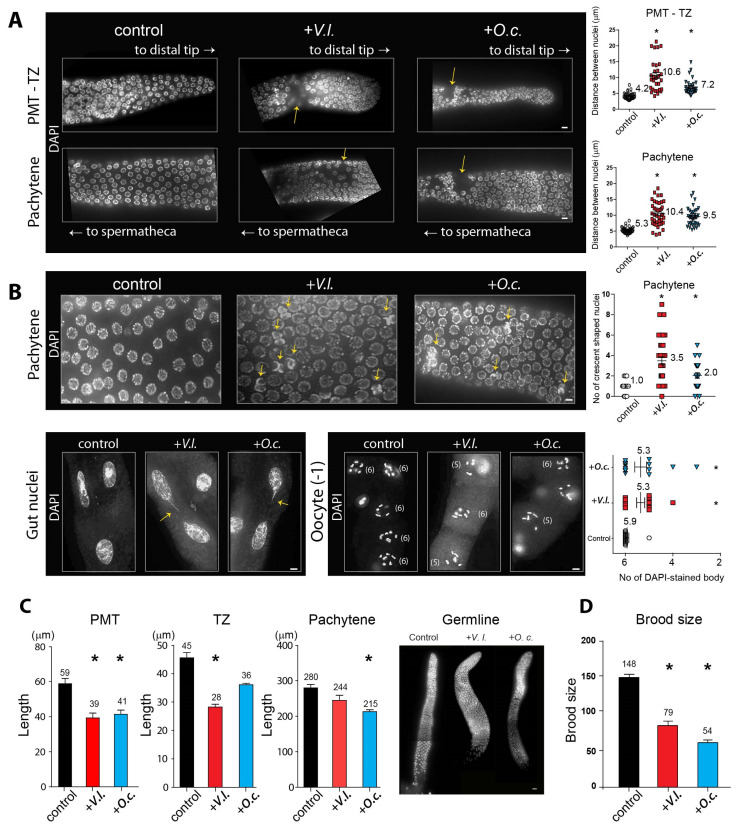
Defective germline development induced by herbal extracts. (**A**) Nuclei arrangement during germline development in control and herb extract-exposed *C. elegans* hermaphrodites. Exposure to *V. lobelianum* (+*V.l.*) and *O. cornuta* (+*O.c.*) extracts resulted in increased gaps indicated by arrows between nuclei in the premeiotic tip–transition zone (PMT-TZ) and pachytene stages. The distances between adjacent nuclei were significantly greater in herb extract-treated worms than in control worms. Asterisks indicate statistical significance according to the two-tailed Mann–Whitney test. Bar = 2 µm. (**B**) Aberrant transition from mitosis to meiosis and chromatin bridge formation. Herbal extract exposure led to aggregates of crescent-shaped nuclei (top row, indicated by arrows) and chromatin bridges in mitotic gut cells (bottom left). Additionally, herb extract-exposed worms exhibited reduced DAPI-stained bodies during diakinesis (bottom right), indicating a faulty DNA recombination process. Asterisks indicate statistical significance according to the two-tailed Mann–Whitney test. Bar = 2 µm. (**C**) Decreased length of PMT, TZ, and pachytene stages. *V. lobelianum* and *O. cornuta* extracts led to reduced lengths of the PMT, TZ, and pachytene stages compared to the control. Asterisks indicate statistical significance according to the two-tailed Mann–Whitney test. Right, the overall shape of the germline upon herb extract exposure. Bar = 10 µm. (**D**) Impaired fertility due to defective germline development. Exposure to herbal extracts resulted in a significant reduction in the number of progenies produced by hermaphrodite worms over a span of four days. *V.I.* and *O.c.* were added to a final concentration of 0.03 μg/mL. Asterisks indicate statistical significance according to the two-tailed Mann–Whitney test. All experiments were performed on *C. elegans* hermaphrodites. Data are presented as the mean ± SEM.

Given that both herb extracts induced defective meiotic progression, we investigated how herbal extracts may affect germline development. We examined adult hermaphrodites that were dissected and stained with DAPI, and we scored the presence of the premeiotic tip (PMT), transition zone (TZ), and pachytene stages. While the nuclei in the control group were precisely ordered during germ cell development, worms exposed to herbal extracts exhibited an increase in gaps between nuclei in the PMT-TZ and pachytene stages, indicating the aberrant progression of germline nuclei (Figure 2A).

The quantification of the distance between adjacent nuclei further confirmed that *V. lobelianum* and *O. cornuta* extracts induced significant spatial disorganization of the nuclei both in the PMT-TZ and pachytene stages (Figure 2A). In the PMT-TZ stage, the distances were 4.2 vs. 10.6 µm in the control and +*V.l.*, *p* < 0.0001; and 4.2 vs. 7.2 µm in the control and +*O.c.*, *p* < 0.0001. In the pachytene stage, the distances were 5.3 vs. 10.4 µm in the control and +*V.l.*, *p* < 0.0001; and 5.3 vs. 9.5 µm in the control and +*O.c.*, *p* < 0.0001, as determined by the two-tailed Mann–Whitney test.

In *C. elegans*, crescent-shaped nuclei indicate the transition from the mitotic to meiotic stages [3,14]. Both herb extracts resulted in aggregates of two or more crescent-shaped nuclei during the pachytene stages, whereas the control group did not, implying that the defective transition from mitosis to meiosis persisted from the TZ to pachytene stages upon herb extract treatment (Figure 2B, top row, 1.0 vs. 3.5 µm in control and +*V.l.*, *p* = 0.0001; 1.0 vs. 2.0 µm in control and +*O.c.*, *p* = 0.0083 by two-tailed Mann–Whitney test).

Consistently, we observed a reduction in the length of the PMT, which is occupied by mitotic nuclei, in *V. lobelianum* and *O. cornuta* compared to the control groups (Figure 2C). The length decreased by 34% (59 vs. 39 µm for the control and +*V.l.*, *p* = 0.0017; 59 vs. 41 µm for the control and +*O.c.*, *p* = 0.0021, determined by a two-tailed *t*-test). Similarly, *V. lobelianum* and *O. cornuta* led to a decrease in the length of the TZ or pachytene stages, where meiotic progression occurs (45 vs. 28 in the control and +*V.l.*, *p* = 0.0003; 45 vs. 36 in the control and +*O.c.*, *p* = 0.076; 280 vs. 244 in the control and +*V.l.*, *p* = 0.076; 280 vs. 215 in the control and +*O.c.*, *p* = 0.0004, as determined by the two-tailed Mann–Whitney test).

In line with larval lethality, we also observed defective mitotic progression in mitotic gut cells (Figure 2B, bottom left). *V. lobelianum* and *O. cornuta* extracts frequently led to the formation of a chromatin bridge, which manifests as a string of chromatin connecting two segregating chromosomes during anaphase or linking daughter nuclei in cytokinesis. In contrast, the untreated control group did not exhibit such a chromatin bridge. This finding indicates the failure to eliminate replication or recombination intermediates [15,16].

During the diakinesis stage of the germline, six bivalents held together by chiasmata, corresponding to six pairs of homologous chromosomes, become visible [3]. However, exposure to the herb extracts failed to form six pairs of homologous chromosomes, resulting in five DAPI-stained bodies in both the *V. lobelianum* and *O. cornuta* groups, indicative of defective DNA repair (Figure 2B, bottom right; see [17]). The counts were 5.9 vs. 5.3 in the control and +*V.l.*, *p* = 0.0045; and 5.9 vs. 5.3 in the control and +*O.c.*, *p* = 0.0035.

Given the reduction in survival exhibited by the herbal extracts, we conducted further tests to determine whether the defective germline progression resulted in a reduced brood size. We counted the number of progenies laid by single hermaphrodite worms, starting from L4 and over a span of four days. We found that both extracts resulted in a significant reduction in fertility, further supporting the idea of defective meiotic development induced by the two herb extracts (Figure 2D). The counts were 148 vs. 79 in the control and +*V.l.*, *p* < 0.0001; 148 vs. 54 in the control and +*O.c.*, *p* < 0.0001).

These observations illustrate how herbal extracts disrupt the orderly progression of the germline, ultimately culminating in reduced fertility. Exposure to these herbal extracts disrupts the usual progression, leading to an increase in gaps between nuclei during the PMT-TZ and pachytene stages. Additionally, the extracts result in aggregates of crescent-shaped nuclei, indicative of a faulty mitotic-to-meiotic transition. This is also supported by a reduction in the PMT, TZ, and pachytene stages, further affecting meiotic progression. Chromatin bridges in mitotic gut cells indicate defective mitotic progression, while the inability to form six pairs of homologous chromosomes during diakinesis suggests faulty DNA repair. These cumulative defects ultimately lead to a reduction in brood size, underscoring the adverse impact of herbal extracts on germline development and fertility in *C. elegans*. In summary, our observations compellingly suggest that *V. lobelianum* and *O. cornuta* extracts disrupt proper germline development and mitotic cell growth, consequently leading to compromised fertility.

### 2.5. Herbal Extracts Activate DNA Damage Checkpoint Pathways: ATM, ATR, CHK1, and Apoptosis

**Figure 3:**
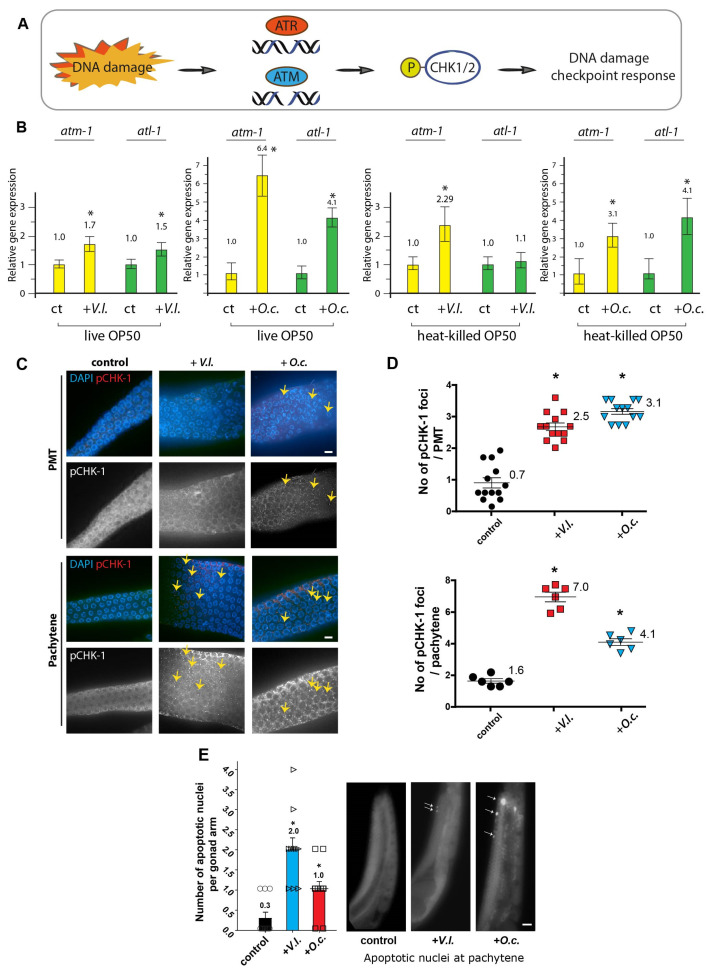
Exposure to herbal extracts of *V. lobelianum* and *O. cornuta* activates the DNA damage response pathway in *C. elegans*, resulting in increased levels of key DNA damage checkpoint components and phosphorylated CHK-1. (**A**) ATM and ATR kinases collaboratively regulate downstream CHK-1 processes in the intricate DNA damage response pathway, playing essential roles in DNA damage repair. (**B**) Exposure to herbal extracts leads to the increased expression of ATM-1 and ATL-1, validating the activation of the DNA damage response pathway. Gene expression levels between live and autoclaved OP50 were comparable, indicating that the nematocidal effects attributed to the herbal extracts may not be influenced by bacterial metabolism. Asterisks indicate statistical significance according to the two-tailed Mann–Whitney test. (**C**) Elevated pCHK-1 foci observed in germlines underscore the active DNA damage response following exposure to herbal extracts, confirming checkpoint activation. Bar = 2 µm. (**D**) Quantification of the number of pCHK-1 foci presented in (**C**). Asterisks indicate statistical significance according to the two-tailed Mann–Whitney test. (**E**) The pachytene stage revealed an increased level of apoptosis, suggesting that unrepaired DNA damage triggers checkpoint activation and subsequent apoptosis in the germline. *V.I.* and *O.c.* were added to a final concentration of 0.03 μg/mL. Asterisks indicate statistical significance according to the two-tailed Mann–Whitney test. Bar = 20 µm.

The DNA damage response is a signaling pathway that coordinates cellular reactions to DNA lesions, triggering a series of responses, such as DNA damage repair, apoptosis, and cell cycle arrest [18,19]. This intricate pathway is regulated by two kinases, ATM and ATR, each with distinct DNA damage specificities. Often, they collaborate to modulate the downstream processes of CHK1 (Figure 3A; see [20]). ATM and ATR can both activate CHK1 either directly or indirectly through intermediate kinases. The activation of CHK1 subsequently initiates downstream events that promote DNA repair, halt cell cycle progression, and uphold genome stability in the face of DNA damage or replication stress.

The defects in DNA damage repair indicate that the herb extracts trigger the activation of the DNA damage checkpoint (Figure 2B). To corroborate the heightened expression of DNA damage checkpoint response pathways, we assessed the levels of ATM-1 (homolog of mammalian ATM), ATL-1 (homolog of mammalian ATR), and pCHK-1 (homolog of mammalian CHK1), the active form of CHK-1. Upon exposure to *V.l.*, there was an increase in the expression of *atm-1* and *atl-1* (Figure 3B), with a 1.7-fold and 1.5-fold induction, respectively (*p* = 0.0026 and *p* = 0.0007). Similarly, *O. cornuta* treatment led to elevated expression of these two pivotal DNA damage checkpoint components, signifying the activation of the DNA damage response due to herbal treatment (with a 6.4-fold induction in *atm-1* and a 4.1-fold induction in *atl-1* expression, *p* = 0.00022 and *p* = 0.00029, respectively).

In line with the mRNA expression profile, elevated levels of pCHK-1 foci were observed in the pachytene stage of germlines, indicating an active DNA damage response following exposure to herbal extracts (Figure 3C; see [19,21]). The two herb extracts exhibited an induction in the PMT and/or pachytene stage, further confirming the activation of the DNA damage checkpoint and CHK-1 phosphorylation (Figure 3C,D, 0.7 vs. 2.5 in PMT of *V. lobelianum*, *p* = 0.0121; 0.7 vs. 3.1 in PMT of *O.c.*, *p* = 0.0072; 1.6 vs. 7.0 in pachytene of *V. lobelianum*, *p* = 0.0004; 1.6 vs. 4.1 in pachytene of *O.c.*, *p* = 0.0008).

Moreover, we investigated whether the observed nematocidal phenotypes were contributed by the secondary metabolites produced by *E. coli* OP50. To test this hypothesis, we fed *C. elegans* with heat-killed OP50 and compared its gene expression level to that of live OP50-fed worms. No discernible differences in gene expression were observed between autoclaved and live OP50 in both herbs, indicating that the nematocidal phenotypes observed with herbal extracts were not a result of bacterial metabolism (Figure 3B).

Since unrepaired DNA intermediates can result in apoptosis in pachytene nuclei [21,22], we further investigated DNA damage-induced apoptosis in the germline. In comparison to the untreated control group, which rarely displayed nuclei highlighted by acridine orange staining, both *V. lobelianum* and *O. cornuta* treatments showed an increase in the number of acridine orange-stained nuclei during pachytene (Figure 3E, 0.3 vs. 2.0 in control and *V.I.*, *p* < 0.0001; 0.3 vs. 1.0 in control and *O.c.*, *p* = 0.0150).

Collectively, our findings demonstrate that exposure to herbal extracts heightened the expression of key components in the ATM/ATR-dependent DNA damage checkpoint pathway and elevated levels of phosphorylated CHK-1, signifying activation of the DNA damage response. These observations strongly indicate that unrepaired DNA damage persists and triggers the DNA damage checkpoint, ultimately leading to increased apoptosis in the *C. elegans* germline. Altogether, the herbal extracts of *V. lobelianum* and *O. cornuta* activate the DNA damage response pathway in *C. elegans*, leading to an increased level of DNA damage-mediated apoptosis.

### 2.6. LC–MS Analysis Identified Anticancer Compounds

**Figure 4:**
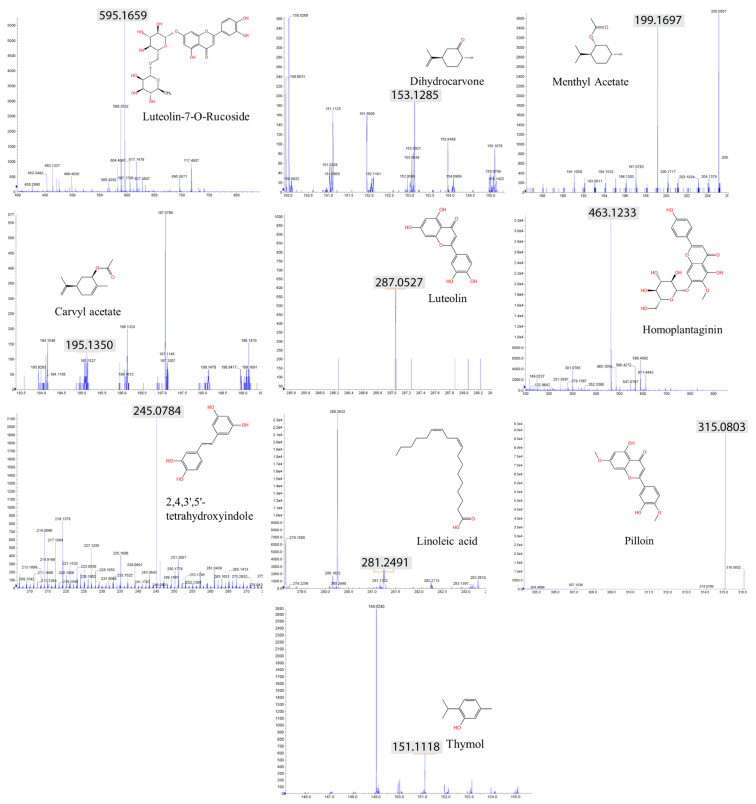
Spectrum of phytochemicals identified in both *V. lobelianum* and *O. cornuta.* Ten compounds were found in both herb extracts. Gray marking and the numbers indicate the predicted fragmentation of compounds provided by Analyst 1.6, an in silico analysis tool. The *x*-axis in an LC–MS graph represents the mass-to-charge ratio (m/z), which indicates the size and charge of ions. The *y*-axis represents the intensity in counts per second (CPS), showing how many ions of a particular m/z are detected.

Herbal extracts encompass a broad spectrum of plant chemicals. To gain insights into the specific compounds contributing to the DNA damage pathway, we conducted an LC–MS (liquid chromatography–mass spectrometry) analysis to identify the biologically active constituents of these extracts. Our analysis of the herbal extract from *V. lobelianum* and *O. cornuta* revealed the presence of 19 and 13 compounds, respectively. Among the major components identified were flavonoids and terpenoids (as shown in Table 1, Figure 4, and Supplemental Figure S1).

The types of phytochemicals are highlighted with different colors. *Veratrum lobelianum* extracts contained 19 identified phytochemicals, while *Onobrychis cornuta* extracts contained 13.

Common compounds identified in both extracts included luteolin-7-O-ructoside, thymol, dihydrocarvone, carvacrol acetate, methyl acetate, luteolin, 2,4,3′,5′-tetrahydroxystilbene, pilloin, linoleic acid, and homoplantain. Additionally, the extract of *V. lobelianum* contained resveratrol, diosmetin, ferruginol, tilianin, vitexin, vitexin-2″-O-rhamnoside, vitexin-4″-O-glucoside, naringin, and jervine, while the extract of *O. cornuta* contained sugiol, dihydrotanshinone I, aucubin, and paclitaxel. The list of detected compounds is provided in Table 1 and Supplementary Figure S1.

### 2.7. V. lobelianum and the Hedgehog Pathway

We hypothesized that the compounds present in the herbs may contribute to the observed phenotypes in the herbal extracts. Jervine, a primary alkaloid found in *V. lobelianum* [23,24], has been identified as an inhibitor of the Hedgehog pathway in nasopharyngeal carcinoma—a crucial cellular pathway involved in cell growth, differentiation, and tissue formation [25,26,27].

**Figure 5:**
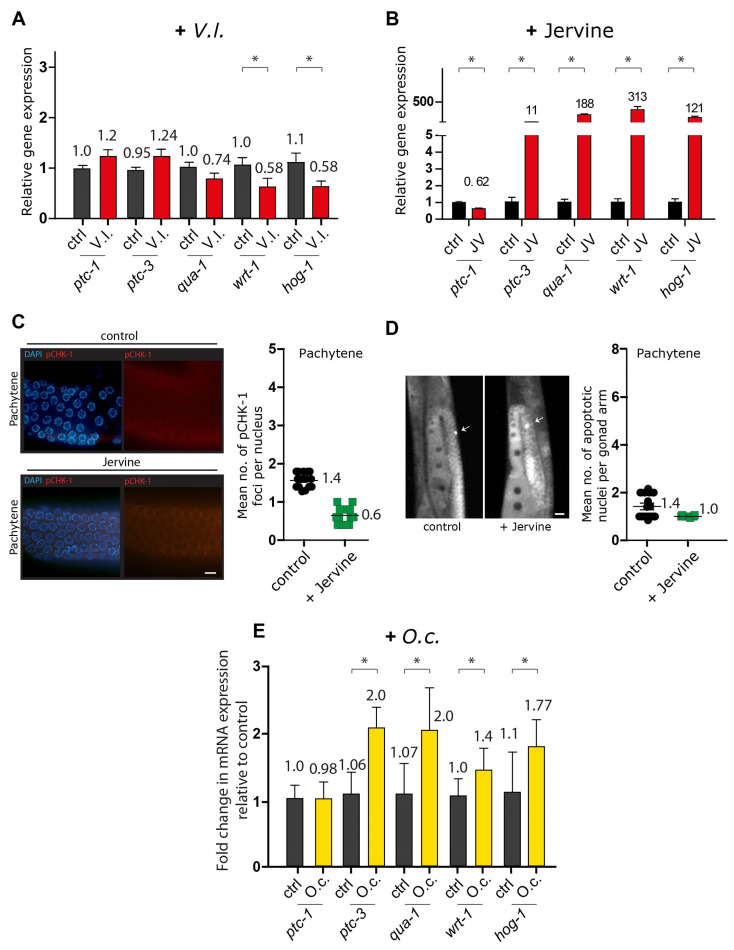
mRNA expression profiles and effects of herbal extracts on DNA damage checkpoint activation, apoptosis, and hedgehog pathway components. (**A**) mRNA expression profile of hedgehog signaling components in *C. elegans* treated with *V. lobelianum* extract. *wrt-1* and *hog-1* were significantly downregulated. (**B**) Upregulation of the hedgehog signaling pathway when jervine was used alone. (**C**) Jervine extract did not increase the number of pCHK-1 foci (1.4 vs. 0.6 in control and +jervine, *p* < 0.0001). Bar = 2 µm. (**D**) *V. lobelianum* extract did not induce apoptosis (1.4 vs. 1.0 in control and +jervine, *p* = 0.1077). Bar = 20 µm. (**E**) *O. cornuta* extract led to the upregulation of the hedgehog pathway components *wrt-1*, *hog-1*, *ptc-3*, and *qua-1*, distinct from *V.l.* extract. *V.I.* and *O.c.* were added to a final concentration of 0.03 μg/mL. The concentration of Jervine was 20 μM. Asterisks indicate statistical significance according to the two-tailed Mann–Whitney test.

Consistent with findings in human carcinoma studies, the mRNA expression profile indicated that two orthologs of Hedgehog signaling components in *C. elegans* were significantly downregulated upon treatment with the *V. lobelianum* extract (Figure 5A; see [28,29]). This suggests that inhibited Hedgehog signaling might trigger DNA damage checkpoint activation, followed by germline apoptosis (Figure 3C–E). Specifically, *wrt-1* expression showed a change of 1.06 vs. 0.58 in the control vs. *V. lobelianum*, with a significance of *p* = 0.0412, and *hog-1* expression displayed a change of 1.11 vs. 0.58 in the control vs. *V. lobelianum*, with a significance of *p* = 0.0379. However, surprisingly, when jervine was used alone but not as part of the herb extract, it led to the upregulation of the hedgehog signaling pathway (Figure 5B). This indicates a different effect compared to its use within the herb extract. Indeed, unlike the *V. lobelianum* extract, jervine per se did not increase the level of pCHK-1 foci or apoptosis (Figure 5C, 1.4 vs. 0.6, *p* < 0.0001; Figure 5D, 1.4 vs. 1.0, *p* = 0.1077). These observations collectively suggest that the *V. lobelianum* extract induces the DNA damage checkpoint and apoptosis in a manner independent of jervine in the *C. elegans* germline, while also compromising the hedgehog pathway.

### 2.8. O. cornuta and the MAPK Kinase Pathway

Similar to *V. lobelianum*, *O. cornuta* extracts triggered the activation of DNA damage checkpoints, induction of germline apoptosis, and defects in germline development (Figure 2 and Figure 3). However, a notable distinction emerged: *O. cornuta* extract led to the upregulation of several components within the hedgehog pathway, such as *wrt-1* and *hog-1* expression, which were downregulated in the *V. lobelianum* extracts. This suggests distinct mechanisms between the two herbal extracts (Figure 5E, 1.0 vs. 1.4 in control and +*O.c.* for *wrt-1* expression, *p* = 0.0040; 1.1 vs. 1.77 in control and +*O.c.* for *hog-1* expression, *p* = 0.0037; 1.06 vs. 2.0 in control and +*O.c.* for *ptc-3* expression, *p* < 0.0001; 1.07 vs. 2.0 in control and +*O.c.* for *qua-1* expression, *p* = 0.0005). Consequently, we screened for potential pathways whose expression levels were altered upon exposure to *O.c.*

The mitogen-activated protein kinase (MAPK) pathway is a critical signaling cascade that regulates various cellular processes, including cell proliferation, differentiation, and survival. It involves a series of protein kinases that activate each other sequentially through phosphorylation events. In germline development, the MAPK pathway plays a pivotal role in modulating the progression of germline nuclei through different meiotic stages, ultimately leading to the production of haploid gametes [30,31].

**Figure 6:**
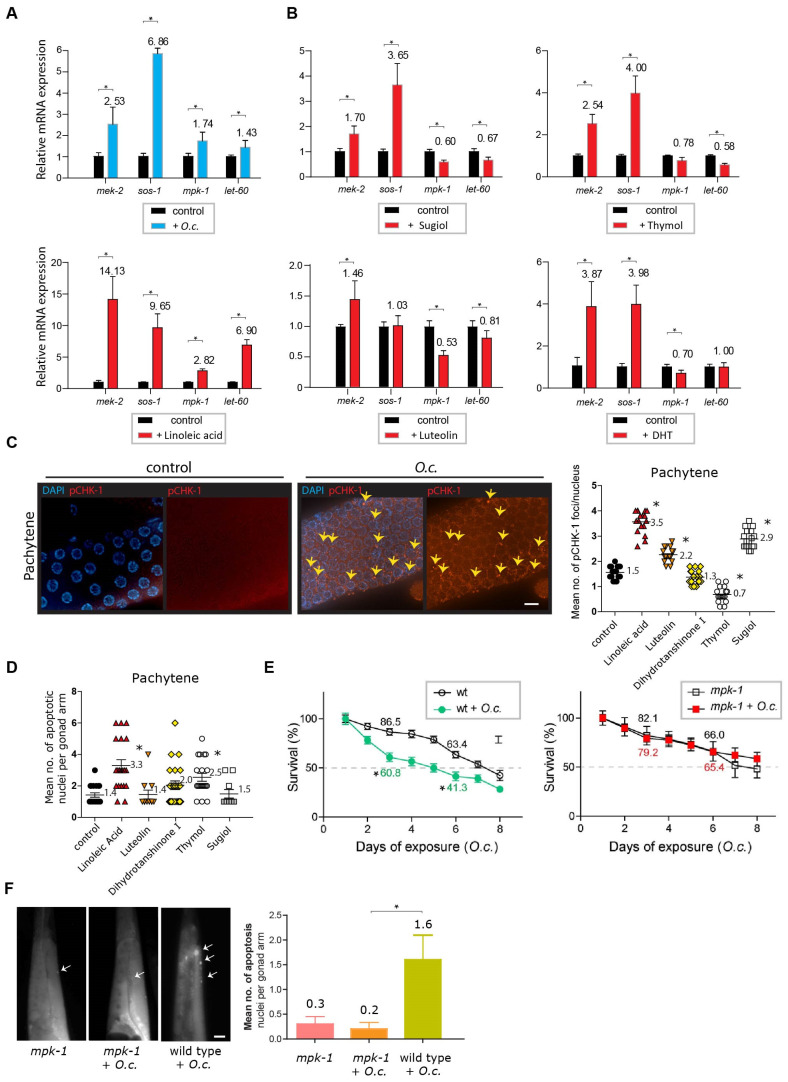
MAPK pathway activation and its role in response to *O. Cornuta* treatment. (**A**) Induction of the MAPK pathway components *mek-2* and *sos-1* upon *O. cornuta* treatment. *mek-2* expression increased by 2.53-fold (*p* = 0.0006), and *sos-1* expression increased by 6.86-fold (*p* = 0.0002) compared to the control. Additionally, *mpk-1* and *let-60* expression was induced by 1.74-fold (*p* = 0.0003) and 1.43-fold (*p* = 0.0003), respectively. Asterisks indicate statistical significance according to the two-tailed Mann–Whitney test. (**B**) Activation of the MAPK pathway by individual compounds in *O. cornuta.* For instance, sugiol treatment increased *mek-2* expression by 1.7-fold (*p* = 0.0002) and *sos-1* expression by 3.65-fold (*p* = 0.0002). Thymol treatment resulted in a 2.54-fold increase in *mek-2* expression (*p* = 0.0009) and a 4.0-fold increase in *sos-1* expression (*p* = 0.0009). Intriguingly, linoleic acid induced all four MAPK components, while other compounds induced one or two genes. Various *O. cornuta* compounds (sugiol, thymol, linoleic acid, luteolin, and DHT) altered MAPK pathway expression. DHT, dihydrotanshinone. (**C**) Linoleic acid-induced DNA damage checkpoint activation. Increased pCHK-1 foci during the pachytene stage (*p* < 0.0001). Arrows indicate pCHK-1 foci. Asterisks indicate statistical significance against the control group. Bar = 2 µm. (**D**) Linoleic acid-triggered apoptosis in germline nuclei similar to *O. cornuta* treatment (*p* < 0.0001). Other *O. cornuta* compounds did not show a simultaneous increase in both pCHK-1 foci and apoptosis. Asterisks indicate statistical significance against the control group. (**E**) The role of the MAPK pathway in the *O. cornuta* response validated by the *mpk-1(ga111)* mutant strain. Reduced survivability was reversed in the mutant (*p* = 0.4256 on day 5, *p* = 0.4208 on day 10 by the two-tailed *t*-test). Relative survivability is presented. Asterisks indicate statistical significance against the control group. (**F**) Suppressed apoptosis in the *mpk-1 (ga111)* mutant, supporting MAPK-dependent DNA damage pathway activation and survivability (*p* = 0.0173 in *mpk-1* +*O.c.* and wild type +*O.c.*). Thymol (2 mM), dihydrotanshinone I (5 μM), luteolin (20 μM), linoleic acid (0.3 μM), and sugiol (10 μM) were employed in a qPCR study. *O.c.* extracts were introduced at a final concentration of 0.03 μg/mL Please refer to the Section 4 for details. Arrows indicate nuclei undergoing apoptosis. Asterisks indicate statistical significance against the control group. Bar = 20 µm.

Surprisingly, the MAPK pathway components *mek-2* and *sos-1* were consistently induced upon treatment with *O. cornuta*, indicating their contribution to the response following *O. cornuta* treatment (Figure 6A, 2.53-fold induction over the control in *mek-2* expression, *p* = 0.0006; 6.86-fold induction in *sos-1* expression, *p* = 0.0002 by two-tailed Mann–Whitney test). Likewise, other players in the MAPK pathway, such as *let-60* (Ras) and *mpk-1* (MAP kinase), were also significantly induced, further validating the activation of the MAPK pathway in response to *O. cornuta* treatment (1.74-fold induction over the control in *mpk-1* expression, *p* = 0.0003; 1.43-fold induction in *let-60* expression, *p* = 0.0003).

Since *O. cornuta* comprises multiple anticancer compounds, we further tested how these compounds in *O. cornuta* function in DNA damage repair. Intriguingly, all five compounds led to the upregulation of *mek-2*, and four of them induced the expression of *sos-1*, suggesting that a majority of these compounds are involved in activating the MAPK pathway (Figure 6B). For instance, under sugiol treatment, *mek-2* expression increased 1.7-fold over the control (*p* = 0.0002), and *sos-1* expression rose 3.65-fold (*p* = 0.0002). Likewise, with thymol treatment, *mek-2* expression showed a 2.54-fold increase over the control (*p* = 0.0009), and *sos-1* expression increased 4-fold (*p* = 0.0009).

Provocatively, linoleic acid induced all four MAPK components, while the other four compounds reduced one or two genes. This implies an unequal contribution of these compounds to the phenotypes in the *O. cornuta* extract (Figure 6B). Specifically, we observed significant increases: 14.13-fold in *mek-2* (*p* = 0.0016), 9.65-fold in *sos-1* (*p* = 0.0007), 2.82-fold in *mpk-1* (*p* = 0.0002), and 6.9-fold in *let-60* expression (*p* = 0.0003).

Given that the expression pattern of linoleic acid is similar to that of *O. cornuta* extracts across four MAPK genes, we explored whether linoleic acid could induce the meiotic defects and activate DNA damage checkpoints observed in *O. cornuta* treatment. Notably, linoleic acid treatment prompted the activation of the DNA damage checkpoint, as evidenced by the increased presence of pCHK-1 foci during the pachytene stage (Figure 6C, 1.5 vs. 3.5 in control and linoleic acid, *p* < 0.0001). This activation of the DNA damage checkpoint consequently triggered apoptosis in germline nuclei, mirroring the observations from *O. cornuta* treatment (Figure 6D, 1.4 vs. 3.3 in control and linoleic acid, *p* < 0.0001). In contrast, among the other four compounds extracted from *O. cornuta*, none demonstrated a simultaneous increase in both pCHK-1 foci and apoptosis. This observation suggests that the phenotypic effects present in *O. cornuta* are uniquely represented by linoleic acid.

The elevated expression of multiple MAPK pathway components suggests a pivotal role for the MAPK pathway in response to *O. cornuta* treatment. We further examined whether the MAPK pathway is indeed responsible for the phenotypes exhibited upon *O. cornuta* exposure. To explore this connection, we employed the *mpk-1(ga111)* mutant strain, which exhibits a potential deficiency in MEK activation in the context of *O. cornuta* exposure [32]. In alignment with our mRNA expression data, defective MEK activation reversed the reduction in survivability, highlighting the substantial involvement of the MAPK pathway in the response to *O. cornuta* treatment (Figure 6E). While *O. cornuta* treatment significantly decreased the survival rate of wild-type worms (86.5 vs. 60.8 on day 3, *p* = 0.0015; 63.4 vs. 41.3 on day 6, *p* = 0.0077), the *mpk-1* mutants showed no marked difference compared to the control groups (82.1 vs. 79.2 on day 3, *p* = 0.3962; 66.0 vs. 65.4 on day 6, *p* = 0.4799 by the two-tailed *t* test).

Moreover, this mutant exhibited a suppressed level of apoptosis, thus further supporting the idea of MAPK pathway-dependent DNA damage pathway activation and survivability upon *O. cornuta* exposure (Figure 6F, 0.2 vs. 1.6 in *mpk-1(ga111)* +*O.c.* and wild type/N2 +*O.c.*, *p* = 0.0173). All of these observations suggest that the MAPK pathway is indispensable for proper survival; therefore, losing MEK activation eliminates the reduction in survival. In summary, our data collectively demonstrated that various constituents of the *O. cornuta* extract enhance MAPK pathway elements and impact worm survival through this pathway. Notably, linoleic acid emerges as a significant player in this intricate process.

## 3. Discussion

This study leveraged *C. elegans* to assess the potential nematocidal toxicity of herbal extracts and elucidated their roles in DNA damage repair and checkpoint responses. A screen of 316 herb extracts identified *O. cornuta* and *V. lobelianum* as activators of DNA damage checkpoint responses, DNA damage apoptosis, HIM, defective meiotic progression, and reduced survival rates. Strikingly, while *V. lobelianum* downregulated hedgehog expression, *O. cornuta*, containing numerous anticancer compounds, upregulated the MAPK pathway, suggesting that distinct pathways mediate the reduced survival and DNA damage checkpoint response. Moreover, defects in the MAPK pathway abolished the phenotypes exhibited in *O. cornuta* exposure (Figure 6F), supporting the idea that MAPK-dependent survivability and DNA damage checkpoint activation play a crucial role in *O. cornuta* treatment.

### 3.1. V. lobelianum

*Veratrum lobelianum* is a robust herbaceous plant found in Northeast Asia, Central Europe, and North America that is known for its toxicity in humans and animals [33]. The alkaloid jervine, abundant in *V. lobelianum*, exhibits antitumor effects and influences DNA damage repair in nasopharyngeal carcinoma cells [24,27,34]. Our experiments, using jervine-containing *V. lobelianum* extract, activated the DNA damage checkpoint by downregulating hedgehog signaling, suggesting a complex relationship (Figure 3 and Figure 4). Despite jervine’s role, the observed phenotypes in *V. lobelianum* may not solely derive from jervine, as it both downregulates and upregulates Hedgehog expression and does not induce pCHK-1 or DNA damage-induced germline apoptosis (Figure 5B–D).

The observed discrepancy may be attributed to different organisms: humans vs. nematodes. However, it is worth noting that a similar discrepancy has also been reported in other mammalian studies. For instance, research has shown that *Veratrum album* extract can protect against radiotherapy-induced gastrointestinal toxicity [35], indicating that herbal extracts containing jervine may have distinct roles compared to jervine alone. With over one hundred alkaloids in Veratrum species, other compounds present in *V. lobelianum* may contribute to nematocidal toxicity, including protoveratrine A and B, which have been implicated in conditions such as arterial hypertension, sleep apnea, and emetic responses [33,36,37]. Further studies are needed to fully understand the impact of alkaloids on nematodes or humans, as well as the collaborative actions within *V. lobelianum* that may lead to synergistic nematode toxicity. Comparative studies on alkaloid combinations and concentrations have the potential to optimize applications such as cancer treatment.

### 3.2. O. cornuta

The flowers and seeds of *Onobrychis cornuta* (L.) Desv. (Fabaceae), native to Iran–Turan and Turkey mountains, are used in traditional medicine [38,39]. This plant is known for its resilience to high temperatures, aridity, diseases, and wireworms like *Dipsosphecia scopigera* and *Sphenoptera carceli*, with an unknown mechanism.

In parallel with *V. lobelianum*, *O. cornuta* extracts resulted in diminished %survival and %adult rates (Figure 2B) and exhibited impaired meiotic progression (Figure 5). The notable increase in pCHK-1 foci count and elevated apoptosis levels in the pachytene germline indicate an apoptotic pathway triggered by DNA damage. These findings collectively underscore the potential of *O. cornuta* extracts as promising candidates for antitumor drug development.

Remarkably, extracts from *O. cornuta* encompass multiple pro-apoptotic compounds, with nine out of thirteen major compounds implicated in anticancer effects (Table 1). The LC–MS analysis of the *O. cornuta* herb yielded highly significant and promising results, suggesting the presence of numerous compounds with potential anticancer properties. Linoleic acid, a vital omega-6 polyunsaturated fatty acid, recapitulated phenotypes induced by *O. cornuta* exposure, including elevated pCHK-1 foci levels, apoptosis, and MAPK pathway activation. While linoleic acid in isolation may mirror certain characteristics of *O. cornuta* treatment, it might not entirely mirror the extensive impact of the complete herbal extract. The complex interplay among various compounds in the herbal extract, as demonstrated by the *O. cornuta* extract revealing many antitumor compounds, implies that the comprehensive effects induced by the entire extract may not be entirely duplicated by linoleic acid alone.

We acknowledge the importance of a systematic assay to examine linoleic acid’s potential. Controlled experiments, assessing both the individual and combined effects of linoleic acid and other major compounds, will provide valuable insights into its contribution. This approach may shed further light on the therapeutic significance of linoleic acid within the broader context of the herbal extract.

Linoleic acid is crucial for diverse physiological processes, including cell membrane structure and function. Autoxidized linoleic acid has the potential to induce DNA strand breaks [40,41,42,43], but it also acts protectively by mitigating DNA damage and apoptosis caused by substances like palmitic acid [44]. This dual nature underscores the importance of a meticulous analysis to fully comprehend linoleic acid’s intricate impacts on both health and cellular processes.

**Figure 7:**
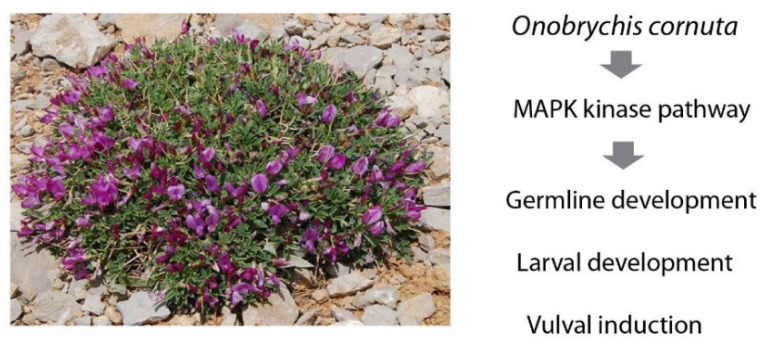
*O. Cornuta* extract induces the upregulation of the MAPK kinase pathway, resulting in germline abnormalities and influencing worm survival. Our observation highlights that not only does *O. cornuta* itself lead to the upregulation of the MAPK pathway, but the compounds within the *O. cornuta* extract also play a significant role in this process. This collective activation of the MAPK pathway underscores its pivotal involvement in influencing germline abnormalities and impacting worm survival. Our observation highlights that not only does *O. cornuta* itself lead to the upregulation of the MAPK pathway, but the compounds within the *O. cornuta* extract also play a significant role in this process. This collective activation of the MAPK pathway underscores its pivotal involvement in influencing germline abnormalities and impacting worm survival. A photograph capturing the exquisite bloom of *Onobrychis cornuta (L.) Desv*. Photographed by Ori Fragman-Sapir (https://flora.org.il/en/plants/ (accessed on 13 December 2023) [46]).

While some hedgehog players were upregulated by *O. cornuta* exposure, treatment with *O. cornuta* induced the expression of MEK-2 and SOS-1, components of the MAPK pathway. SOS-1 serves as a putative guanine nucleotide exchanger for LET-60 Ras [45], facilitating the exchange of GDP for GTP on Ras proteins and activating them. Overexpressing GEF leads to elevated Ras signaling pathway activity. The expression of LET-60/Ras and MPK-1/MAP kinase was also stimulated, further validating the activation of the MAPK pathway due to *O. cornuta* treatment (Figure 6 and Figure 7).

Our findings align with existing knowledge, as the two genes involved in the Let-60/Ras-mediated MAPK pathway play pivotal roles in proper germline development [30]. This underscores the significance of MAPK in *O. cornuta* treatment responses. Notably, gain-of-function *mek-2* alleles induced pleiotropic defects, including embryonic lethality and a multiple vulva (Muv) phenotype, while loss-of-function alleles suppressed the phenotype (Figure 7). Likewise, the *mpk-1 (ga111) mutant*, defective in MEK activation [32], suppressed the reduced survivability and higher level of apoptosis observed under *O. cornuta* exposure, indicating the role of the MAPK pathway in defective germline progression and DNA damage checkpoint response activation (Figure 6E, F). Collectively, our results indicate that *O. cornuta*-induced MAPK pathway overexpression leads to abnormal meiotic development and reduced survivability. Encouragingly, the five major *O. cornuta* compounds upregulate *mek-2* and *sos-1*. Subsequent studies on the MAPK pathway should aim to uncover how *O. cornuta* upregulates this pathway.

### 3.3. V. lobelianum and O. cornuta Herbal Extracts: Balancing Promise and Toxicity

While herbal extracts from *V. lobelianum* and *O. cornuta* show promise as novel antitumor drugs by halting the cell cycle, they also exhibit toxicity to noncancerous cells. For instance, in lambs, Veratrum ingestion causes birth defects, including craniofacial issues [47]. Similarly, our research indicates that both extracts disrupt meiotic processes during germline nuclei development (Figure 2), indicating flaws in meiotic development in *C. elegans* [48,49].

Interestingly, exposure to *O. cornuta* prompts an intriguing rise in the MAPK pathway, leading to hindered meiotic progression. Activating this pathway could offer a valuable tool for the in-depth exploration of cellular signaling mechanisms, thus aiding the identification of therapeutic targets for various diseases.

Our data strongly support the involvement of the MAPK pathway in *O. cornuta*’s effects, further supported by the LC–MS analysis revealing anticancer compounds in *O. cornuta* extracts. Compounds of *O. cornuta* extracts and the O. cornuta extract per se stimulate critical components of the MAPK pathway, reinforcing its role in germline development.

Despite discovering thirteen noteworthy compounds within *O. cornuta*—with nine known for their anticancer properties—the precise ways they affect the MAPK pathway require further elucidation. Comparing *V. lobelianum* and *O. cornuta* extracts highlights the differences in their molecular mechanisms and signaling pathways that eventually produced similar output: DNA damage apoptosis and defective germline development. The use of the mutant strain, which displays reduced MEK activation, provides compelling evidence for the central importance of the MAPK pathway in the abnormal effects induced by *O. cornuta.*

This study presents a compelling array of evidence highlighting the potential antitumor effects stemming from extracts from *O. cornuta* and *V. lobelianum*. These effects can be attributed to their notable influence on critical processes linked to DNA damage repair, activation of DNA damage checkpoint responses, and their skillful modulation of the MAPK pathway. However, the weight of these findings demands a tempered approach, urging us to tread cautiously due to conceivable toxicity concerns in noncancerous cells. The intricate interplay observed among the various compounds within these herbal extracts emphasizes the magnitude of our discoveries. Their multifaceted impact on cellular processes underscores the paramount importance of our findings in the broader landscape of scientific understanding.

The presence of linoleic acid in *O. cornuta* and Jervine in *V. lobelianum* highlights key components contributing to their observed antitumor properties. These findings position both *V. lobelianum* and *O. cornuta* as promising resources for developing potential antitumor drugs, suggesting a potential breakthrough in cancer treatment through further exploration of their constituent elements.

## 4. Materials and Methods

### 4.1. Strains and Alleles

All *C. elegans* strains were cultured at 20 °C under standard conditions, as described, and the N2 Bristol strain was used as the wild type.

### 4.2. Herb Extraction

*Veratrum lobelianum* and *Onobrychis cornuta* were collected in Armenia in May 2006. Plant material was freed of extraneous matter, air-dried in the shade, milled to a coarse powder, and extracted with methanol. The pooled methanol extract was concentrated in vacuo to afford a tarry residue. The methanol extract was dissolved in 90% (aqueous) methanol and extracted with n-hexane. The residual hydroalcoholic phase was freed of the solvent in vacuo, suspended in water, and then sequentially extracted with dichloromethane and n-butanol (n-BuOH) to afford a gross separation into hexane-, dichloromethane-, butanol-, and water-soluble fractions. The resulting extracts were dissolved in DMSO, diluted to a final concentration of 1 mg/mL in DMSO, and subsequently further diluted using M9 buffer to achieve a final concentration of 0.03 µg/mL. Three solvents, namely hexane, butanol, and water, were designated -H, -B, and -A, respectively.

### 4.3. Survival, Larval Arrest/Lethality and HIM

Gravid hermaphrodites were collected from NGM plates to establish synchronized L1-stage larvae, following the protocol outlined in [19,50]. These synchronized worms were then suspended in 180 µL of the herb extract solution and transferred to a 96-well plate. Subsequently, the worms were gently agitated and incubated at 20 °C for 24 h. Phenotypic changes were monitored continuously, extending up to 48 h.

To assess relative survival, the worms’ mobility was monitored after 24 h of incubation. The brood sizes represent the total number of eggs laid by each individual worm during the 4–5 days following the L4 stage. Larval arrest or lethality indicates the percentage of hatched worms that did not survive to reach adulthood. Additionally, the proportion of males in the population (%Him) was calculated, representing the percentage of adult worms that were males.

For each reported generation, an analysis was conducted on the entire progeny of approximately 20 worms for each genotype. Statistical comparisons between different genotypes were carried out using the two-tailed Mann–Whitney test with a 95% confidence interval (C.I.). All experiments were performed in triplicate to ensure reproducibility.

### 4.4. Cumulative Survival

At least 60 L4-stage worms were collected for the assessment of survivability. Chemical solutions were prepared by diluting them with M9 buffer. The concentration of linoleic acid in these solutions was maintained at 0.3 µM, as referenced in [51].

The procedure for the survivability study was adopted from the work of Kim and Colaiacovo [50]. L4 stage worms were gathered and placed in a 24-well plate, along with the appropriately diluted chemical solutions. Incubation was carried out at a temperature of 20 °C overnight. After incubation, the worms underwent three rounds of rinsing with M9 buffer to remove residual chemicals from their exteriors. Subsequent to the rinsing process, the worms were transferred onto NGM plates, with each plate accommodating 20 worms. Three replicates were prepared for each treatment. The survival rates of the worms were monitored for 10 days. On a daily basis, any surviving worms were moved to fresh NGM plates following the counting procedure.

### 4.5. Preparation of Worm Lysates for Mass Spectrometric Analysis

The worm preparation methods used for the LC–MS analysis are described in [52]. Age-matched worms (20 h post-L4) were exposed to M9 buffer with different herb extracts (0.03 µg/mL) at 25 °C for 20 h. After exposure, worms were washed 10 times in M9 buffer and frozen with minimal M9 in liquid nitrogen. The worm pellet was resuspended in lysis buffer (0.5 M sucrose, 25 mM HEPES (pH 7.6), 5 mM EDTA, 0.5% CHAPS, and 0.5% deoxycholic acid). Samples were homogenized and centrifuged to remove worm fragments.

### 4.6. LC–MS Analysis

The identification of compounds was conducted through an LC–MS analysis by Yanbotimes (Beijing, China). The LC–MS/MS method used in this study employed a Shimadzu LC-30A chromatography system with a C18 column (2.2 μm, 2.1 × 100 mm). The column temperature was maintained at 40 °C, and the flow rate was set at 0.2 mL/min. A 2 μL sample was injected into the column. The mobile phase consisted of acetonitrile and 0.1% formic acid solution. The mass spectrometry instrument used was the AB Sciex Triple TOF 5600+. For the positive ionization mode, the ion source voltage was 5500 V, and the ion source temperature was set to 500 °C. The declustering potential (DP) was set at 100 V, collision energy (CE) at 40 eV, and collision energy spread (CES) at 15 eV. Nitrogen gas was used as the nebulizing gas, with 50 PSI for both auxiliary gases 1 and 2 and 40 PSI for the curtain gas. The mass spectrometer performed first-level MS scanning in the range of 100–1500 m/z, followed by second-level MS scanning for peaks with a response greater than 100 cps. The range for second-level MS scanning was also set at 100–1500 m/z, and dynamic background subtraction (DBS) was enabled. For the negative ionization mode, the ion source voltage was set at −4500 V, and all other parameters remained the same as in the positive ionization mode. To ensure accuracy, results were compared with the company’s standard sample database. All compounds presented in Table 1 were identified using this rigorous process.

### 4.7. Monitoring the Growth of E. coli

The growth performance of *E. coli* OP50 in different herb extracts was analyzed via optical density, as described in [53]. To test whether the herb extracts show any antibacterial impact against *E. coli* OP50, the bacterial growth was determined by frequently measuring the optical density (at 600 nm) during exposure to 0.03 µg/mL of each herb extract [54].

### 4.8. Immunofluorescence Staining

Whole-mount preparations of dissected gonads and fixation and immunostaining procedures were carried out as described in [55]. Primary antibodies were used at the following dilutions: rabbit pCHK-1 (1:250, Cell Signaling, Ser345). The secondary antibodies used were Cy3 anti-rabbit (1:300) from Jackson Immunochemicals. Immunofluorescence images were collected at 0.2 μm intervals with an Eclipse Ti2-E inverted microscope and a DSQi2 camera (Nikon, Japan). Photos were taken with a 60× objective combined with 1.5× auxiliary magnification and were subjected to deconvolution, using NIS Elements software ver. 4.0 (Nikon). Partial projections of half nuclei are shown.

### 4.9. Quantitative Analysis of pCHK-1 Foci

Quantitation of pCHK-1 foci was performed as described in [55]. Between five and ten germlines were scored for each treatment. Statistical comparisons between treatments were performed using the two-tailed Mann–Whitney or *t*-test with a 95% confidence interval.

### 4.10. Quantitation of Germline Apoptosis

Germlines of age-matched (20 h post-L4) animals were analyzed by acridine orange staining, as described in [56], utilizing a Nikon Ti2-E fluorescence microscope. Between 20 and 30 gonads were scored for each treatment. Statistical comparisons between treatments were performed using the two-tailed Mann–Whitney test, 95% C.I.

### 4.11. Quantitative Real-Time PCR (qPCR)

cDNA was produced from young hermaphrodite worm RNA extracts, using the ABscript II First synthesis (ABclonal RK20400). Real-time quantitative PCR was carried out using ABclonal 2X SYBR Green Fast mix (Abclonal RK21200). Amplification was conducted in a LineGene 4800 (BIOER FQD48A) with initial polymerase activation at 95 °C for 2 min, followed by 40 cycles of 95 °C for 15 s denaturation and 60 °C for 20 s for annealing and elongation. After 40 cycles, a melting curve analysis was carried out (60 °C to 95 °C) to verify the specificity of amplicons. Tubulin encoding *tba-1* was selected for a reference gene based on *C. elegans* microarray expression data. Each qPCR was repeated independently at least 2–3 times. Concentrations of phytochemicals were utilized for qPCR: thymol at 2 mM [57], dihydrotanshinone I at 5 μM [58], luteolin at 20 μM [59], linoleic acid at 0.3 μM [51], and sugiol at 10 μM [60].

## 5. Conclusions

The study emphasizes the intricate interplay among the compounds in these herbal extracts, highlighting the significance of the discoveries. Key components, such as linoleic acid in *O. cornuta*, are identified as contributing to their observed antitumor properties. The study suggests that these plants could be valuable resources for developing potential antitumor drugs, representing a potential breakthrough in cancer treatment through further exploration of their constituent elements.

## Figures and Tables

**Table 1:** Phytochemical composition of *Veratrum lobelianum* and *Onobrychis cornuta*.

No	Compound	Rt	[M+H]^+^	Fragments	Herbs	Types
1	Luteolin-7-O-Rucoside	12.091	594.1658	286.0477, 301.0713, 463.1224	*V.l.* and *O.c.*	Flavonoids
2	Thymol	20.594	151.1123	77.0408, 91.0574, 105.0714, 107.0540	*V.l.* and *O.c.*	Terpenoids
3	Dihydrocarvone	26.597	153.1276	152.1122	*V.l.* and *O.c.*	Terpenoids
4	Carvyl acetate	17.201	195.1379	153.1283	*V.l.* and *O.c.*	Terpenoids
5	Menthyl Acetate	27.991	199.1694	144.0785, 111.1197, 55.0596, 69.0730	*V.l.* and *O.c.*	Terpenoids
6	Luteolin	1.36	287.0549	168.0056, 140.0109	*V.l.* and *O.c.*	Flavonoids
7	2,4,3′,5′-tetrahydroxystilbene	21.186	245.0784	228.0723, 180.9145, 140.9176	*V.l.* and *O.c.*	Phenolic compounds
8	Pilloin	1.125	315.0803	182.0429	*V.l.* and *O.c.*	Carotenoid
9	Linoleic acid	28.325	281.2475	248.9901, 151.0287	*V.l.* and *O.c.*	Fatty acids
10	Homoplantain	12.761	463.1234	301.0711, 286.0476	*V.l.* and *O.c.*	Alkaloids
11	Resveratrol	23.73	229.0858	151.0394	*V.l.*	Phenolic compounds
12	Diosmetin	12.762	301.0707	286.0476, 168.0044	*V.l.*	Lignin
13	Ferruginol	27.55	287.2376	173.1332, 93.0721	*V.l.*	Prostaglandins
14	Tilianin	18.678	447.1293	294.1053, 259.1366, 105.0362	*V.l.*	Flavonoids
15	Vitexin	12.589	433.1134	286.0474	*V.l.*	Flavonoids
16	Vitexin-2″-O-rhamnoside	10.538	579.1711	301.1366, 285.0770	*V.l.*	Flavonoids
17	Vitexin-4″-O-glucoside	12.091	595.1658	463.1240, 301.0713, 286.0477	*V.l.*	Flavonoids
18	Naringin	13.299	581.1863	273.0768	*V.l.*	Flavonoids
19	Jervine	11.59	426.3007	313.2138	*V.l.*	Alkaloids
20	Sugiol	23.15	301.216	259.1685, 163.0752	*O.c.*	Terpenoids
21	Dihydrotanshinone I	19.354	279.0933	152.1122	*O.c.*	Tanshinones
22	Aucubin	26.472	347.1333	216.9976, 129.0189	*O.c.*	Iridoids
23	Paclitaxel	5.34	297.77	279.0874	*O.c.*	Iridoids

## Data Availability

Data are contained within the article and supplementary materials.

## Supplementary Materials

The following supporting information can be downloaded at: https://www.mdpi.com/article/10.3390/nu16010008/s1, Table S1: Samples IDs in 96-well plates (Note that some samples are redundant); Figure S1: Compounds found in *Veratrum lobelianum* and *Onobrychis cornuta* extracts.
