# Supplementary material for: Exploring the Impact of Onobrychis cornuta and Veratrum lobelianum Extracts on C. elegans: Implications for MAPK Modulation, Germline Development, and Antitumor Properties
Source: Nutrients. 2023 Dec 19;16(1):8. doi: 10.3390/nu16010008 (PMC10780469; doi:10.3390/nu16010008)
Supplement: Supplementary file 1 [file nutrients-16-00008-s001.zip › nutrients-2687836-supplementary.pdf]

Supplemental Table S1. Samples IDs in 96-well plates (Note that some samples are redundant)

	**1**	**2**	**3**	**4**	**5**	**6**	**7**	**8**	**9**	**10**	**11**	**12**
A	1H	2H	3H	4H	5H	6H	7H					
B	1D	2D	3D	4D	5D	6D	7D					
C	1B	2B	3B	4B	5B	6B	7B					
D	1A	2A	3A	4A	5A	6A	7A					

	**1**	**2**	**3**	**4**	**5**	**6**	**7**	**8**	**9**	**10**	**11**	**12**
A	1H	2H	3H	4H	5H	6H	7H	8H	9H	10H	11H	12H
B	1D	2D	3D	4D	5D	6D	7D	8D	9D	10D	11D	12D
C	1B	2B	3B	4B	5B	6B	7B	8B	9B	10B	11B	12B
D	1A	2A	3A	4A	5A	6A	7A	8A	9A	10A	11A	12A
E	13H	14H	15H	16H	17H	18H	19H	20H	21H	22H	23H	24H
F	13D	14D	15D	16D	17D	18D	19D	20D	21D	22D	23D	24D
G	13B	14B	15B	16B	17B	18B	19B	20B	21B	22B	23B	24B
H	13A	14A	15A	16A	17A	18A	19A	20A	21A	22A	23A	24A

	**1**	**2**	**3**	**4**	**5**	**6**	**7**	**8**	**9**	**10**	**11**	**12**
A	25H	26H	27H	28H	29H	30H	31H	32H	33H	34H	35H	36H
B	25D	26D	27D	28D	29D	30D	31D	32D	33D	34D	35D	36D
C	25B	26B	27B	28B	29B	30B	31B	32B	33B	34B	35B	36B
D	25A	26A	27A	28A	29A	30A	31A	32A	33A	34A	35A	36A
E	37H	38H	39H	40H	41H	42H	43H	44H	45H	46H	47H	48H
F	37D	38D	39D	40D	41D	42D	43D	44D	45D	46D	47D	48D
G	37B	38B	39B	40B	41B	42B	43B	44B	45B	46B	47B	48B
H	37A	38A	39A	40A	41A	42A	43A	44A	45A	46A	47A	48A

	**1**	**2**	**3**	**4**	**5**	**6**	**7**	**8**	**9**	**10**	**11**	**12**
A	49H	50H	51H	52H	53H	54H	55H	56H	57H	58H	59H	60H
B	49D	50D	51D	52D	53D	54D	55D	56D	57D	58D	59D	60D
C	49B	50B	51B	52B	53B	54B	55B	56B	57B	58B	59B	60B
D	49A	50A	51A	52A	53A	54A	55A	56A	57A	58A	59A	60A
E	61H	62H	63H	64H	65H	66H	67H	68H	69H	70H	123H	124H
F	61D	62D	63D	64D	65D	66D	67D	68D	69D	70D	123D	124D
G	61B	62B	63B	64B	65B	66B	67B	68B	69B	70B	123B	124B
H	61A	62A	63A	64A	65A	66A	67A	68A	69A	70A	123A	124A

Sample IDs and corresponding herbs

	Genus	***Species***	**Authority**	**Family**	**Part**
**1**	*Ruscus*	*hyrcanus*	Woronow	Asparagaceae	LF+FR
**2**	*Hypericum*	*androsaemum*	L.	Clusiaceae	PL
**3**	*Dryopteris*	*raddeana*	(Fomin) Fomin	Dryopteridaceae	LF
**4**	*Buxus*	*hyrcana*	Pojark.	Buxaceae	RT
**5**	*Buxus*	*hyrcana*	Pojark.	Buxaceae	ST
**6**	*Polygonum*	*hydropiper*	L.	Polygonaceae	PL
**7**	*Solanum*	*dulcamara*	L.	Solanaceae	ST+RT

8	*Solanum*	*dulcamara*	L.	Solanaceae	LF+FL+FR
9	*Carex*	*sylvatica*	Huds.	Cyperaceae	PL
10	*Crataegus*	*microphylla*	K. Koch	Rosaceae	ST
11	*Crataegus*	*microphylla*	K. Koch	Rosaceae	LF
12	*Scrophularia*	*cinerascens*	Boiss.	Scrophulariaceae	ST+RT
13	*Scrophularia*	*cinerascens*	Boiss.	Scrophulariaceae	LF+FL+FR
14	*Zelkova*	*carpinifolia*	(Pall.) K. Koch	Ulmaceae	ST
15	*Astragalus*	*igniarius*	Popov	Fabaceae	ST+RT
16	*Chaerophyllum*	*bulbosum*	L.	Apiaceae	PL
17	*Pimpinella*	*peregrina*	L.	Apiaceae	PL
18	*Spiraea*	*crenata*	L.	Rosaceae	PL
19	*Hypericum*	*scabrum*	L.	Clusiaceae	PL
20	*Aethionema*	*armenum*	Boiss.	Brassicaceae	PL
21	*Achillea*	*biebersteinii*	Afan.	Asteraceae	PL
22	*Eremostachys*	*macrophyll*	Montbret &	Lamiaceae	PL
23	*Glycyrrhiza*	*glabra*	L.	Fabaceae	PL
24	*Camphorosma*	*lessingii*	Litv.	Chenopodiaceae	PL
25	*Capparis*	*herbacea*	Willd.	Capparaceae	PL
26	*Astracantha*	*microcephala*	(Willd.)Podlech.	Fabaceae	PL
27	*Chardinia*	*orientalis*	(L.) Kuntze	Asteraceae	PL
28	*Thalictrum*	*minus*	L.	Ranunculaceae	PL
29	*Thymus*	*kotschyanus*	Boiss. & Hohen.	Lamiaceae	PL
30	*Echium*	*russicum*	J. F. Gmel.	Boraginaceae	PL
31	*Galium*	*verum*	L	Rubiaceae	PL
32	*Veratrum*	*lobelianum*	Bernh.	Melanthiaceae	PL
33	*Caltha*	*polypetala*	Hochst.	Ranunculaceae	PL
34	*Mentha*	*longifolia*	(L.) Huds.	Lamiaceae	PL
35	*Scrophularia*	*orientalis*	L.	Scrophulariaceae	PL
36	*Lappula*	*patula*	(Lehm.) Asc	Boraginacea	LF+FL+FR
37	*Galium*	*ruthenicum*	Willd.	Rubiaceae	ST+RT
38	*Oberna*	*multifida*	(Adams) Ikonn.	Caryophyllaceae	PL
39	*Artemisia*	*lercheana*	Weber	Asteraceae	ST+RT
40	*Artemisia*	*lercheana*	Weber	Asteraceae	LF+FL

41	*Echium*	*biebersteinii*	(Lacaita) Dobrocz.	Boraginaceae	ST+RT
42	*Juniperus*	*oblonga*	M. Bieb.	Cupressaceae	RT
43	*Juniperus*	*oblonga*	M. Bieb.	Cupressaceae	LF+FR
44	*Juniperus*	*oblonga*	M. Bieb.	Cupressaceae	ST
45	*Cruciata*	*articulata*	(L.) Ehrend.	Rubiaceae	PL
46	*Symphytum*	*caucasicum*	M. Bieb.	Boraginaceae	PL
47	*Ambrosia*	*artemisiifolia*	L.	Asteraceae	LF+FL
48	*Ambrosia*	*artemisiifolia*	L.	Asteraceae	RT
49	*Ambrosia*	*artemisiifolia*	L.	Asteraceae	ST
50	*Stachys*	*lavandulifolia*	Vahl	Lamiaceae	PL
51	*Zygophyllum*	*fabago*	L.	Zygophyllaceae	PL
52	*Elaeagnus*	*orientalis*	L.	Elaeagnace	PL
53	*Lepidium*	*vesicarium*	L.	Brassicaceae	PL
54	*Rhamnus*	*pallasii*	Fisch. & C. A. Mey.	Rhamnaceae	PL
55	*Atraphaxis*	*spinosa*	L.	Polygonaceae	PL
56	*Onobrychis*	*cornuta*	(L.) Desv.	Fabaceae	PL
57	*Geranium*	*tuberosum*	L.	Geraniacea	PL
58	*Crataegus*	*orientalis*	Pall. ex M. Bieb.	Rosaceae	LF
59	*Primula*	*macrocalyx*	Bunge	Primulaceae	PL
60	*Caccinia*	*macranthe*	(Banks & S	Boraginac	PL
61	*Scutellaria*	*orientalis*	L.	Lamiaceae	PL
62	*Symphytum*	*asperum*	Lepech.	Boraginaceae	PL
63	*Melandrium*	*album*	(Mill.) Garcke	Caryophyllaceae	PL
64	*Cardaria*	*boissieri*	(N. Busch) Soó	Brassicaceae	PL
65	*Euphorbia*	*macroceras*	Fisch. & C.A	Euphorbiac	PL
66	*Amberboa*	*moschata*	(L.) DC.	Asteraceae	PL
67	*Erodium*	*oxyrhynchu*	M. Bieb.	Geraniacea	PL
68	*Anchusa*	*azurea*	Mill.	Boraginaceae	PL
69	*Xerophyllum*	*tenax*	(Pursh) Nutt.	Melanthiaceae	RT+RHZ
70	*Torenia*	*sp.*		Scrophulariaceae	PL
123	*Metrosideros*	*polymnorpha*		Myrtaceae	LF
124	*Artocarpus*	*altilis*		Moraceae	LF

125	*Metrosideros*	*polymorpha*	Myrtaceae	ST
126	*Artocarpus*	*altilis*	Moraceae	ST
127	*Curcuma*	*longa*	Zingiberaceae	RHZ

Supplemental Figure S1. Compounds found in *Veratrum lobelianum* and *Onobrychis cornuta* extracts

Compounds found in *Veratrum lobelianum* and *Onobrychis cornuta* extracts. The x–axis in an LC–MS graph represents the mass–to–charge ratio (m/z). The y–axis represents intensity in counts per second (cps).

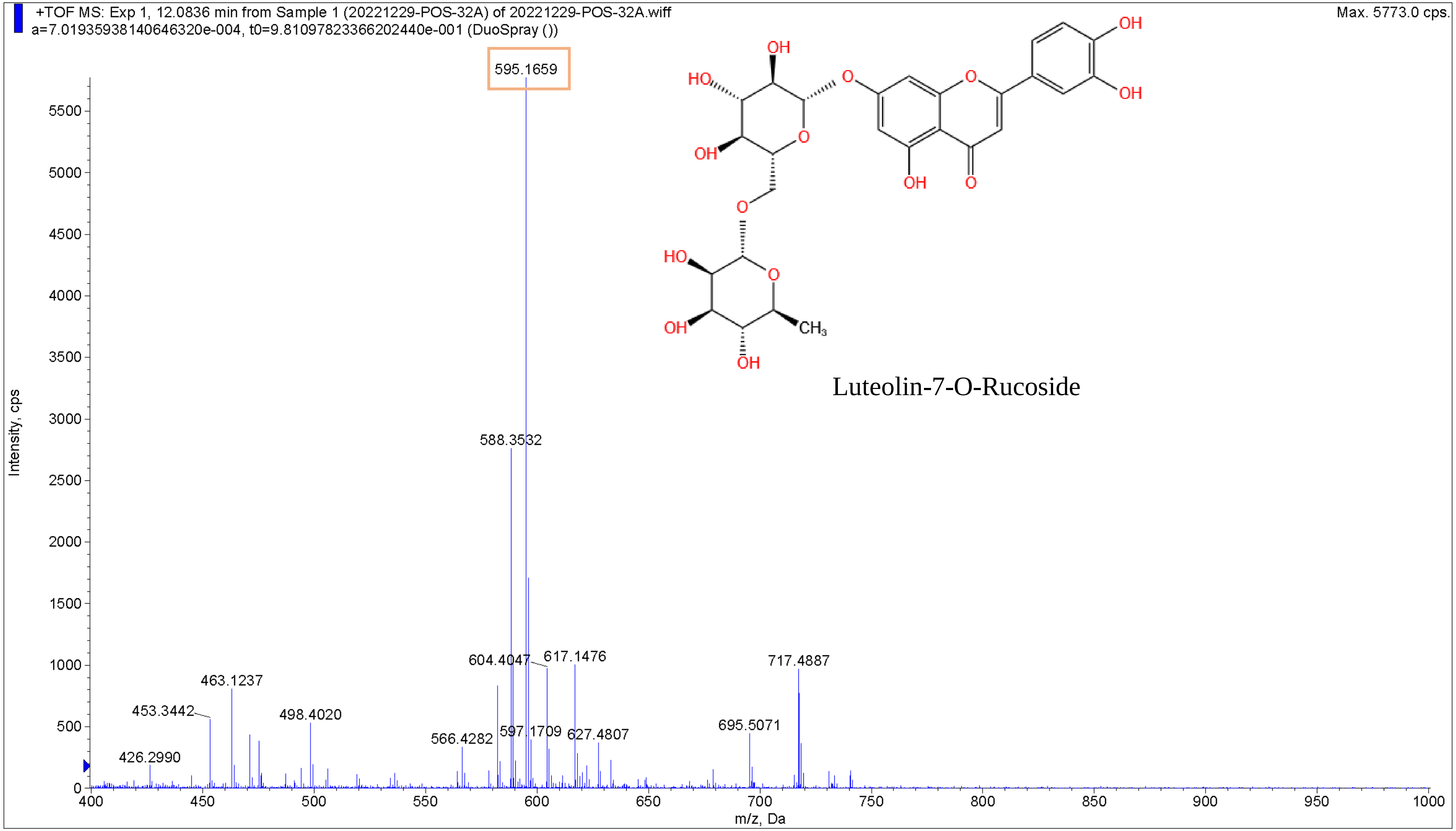

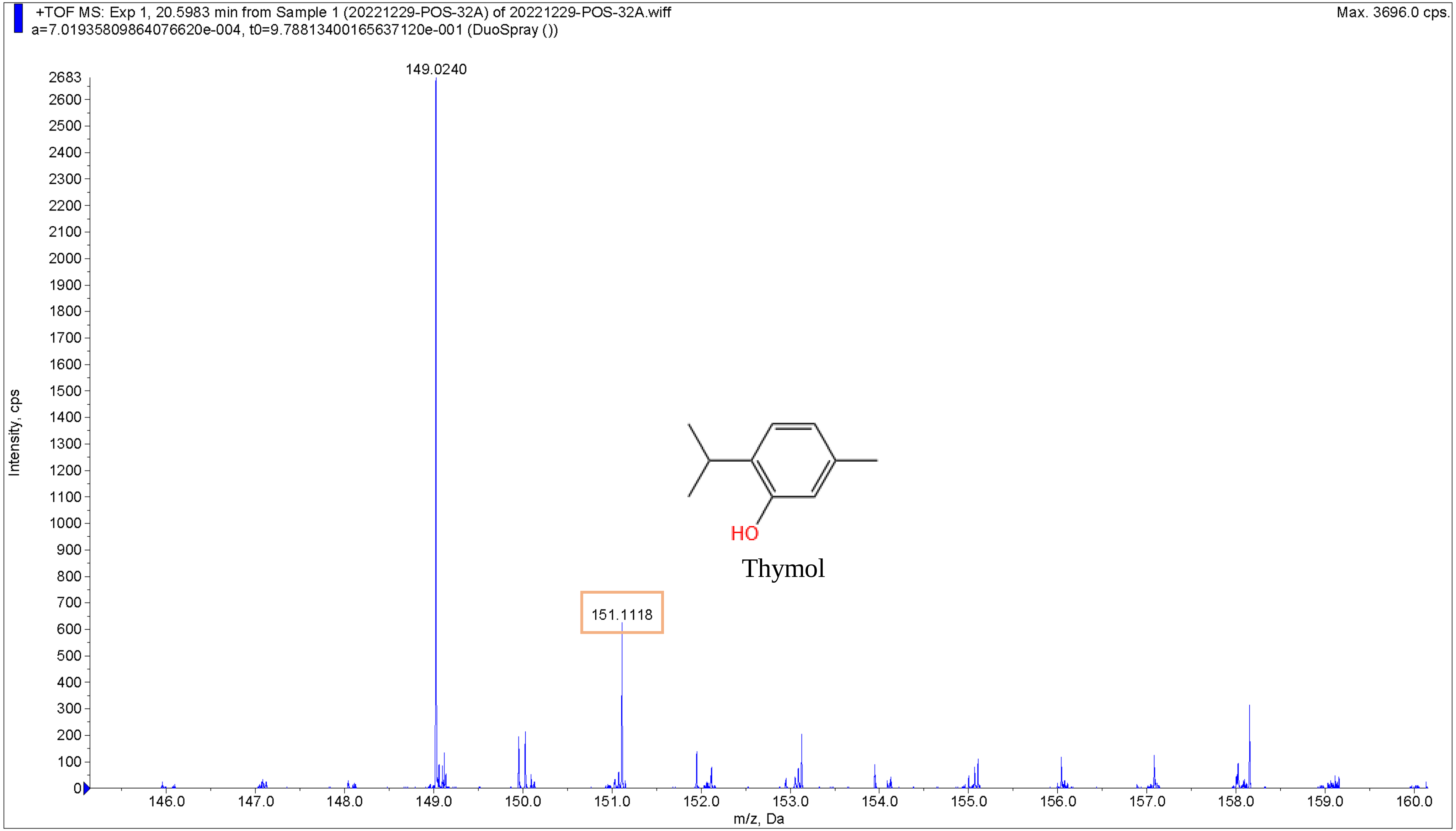

+TOF MS: Exp 1, 20.5983 min from Sample 1 (20221229-POS-32A) of 20221229-POS-32A.wiff
a=7.01935809864076620e-004, t0=9.78813400165637120e-001 (DuoSpray ())
Max. 3696.0 cps.
149.0240
151.1118
HO
Thymol
Intensity, cps
m/z, Da

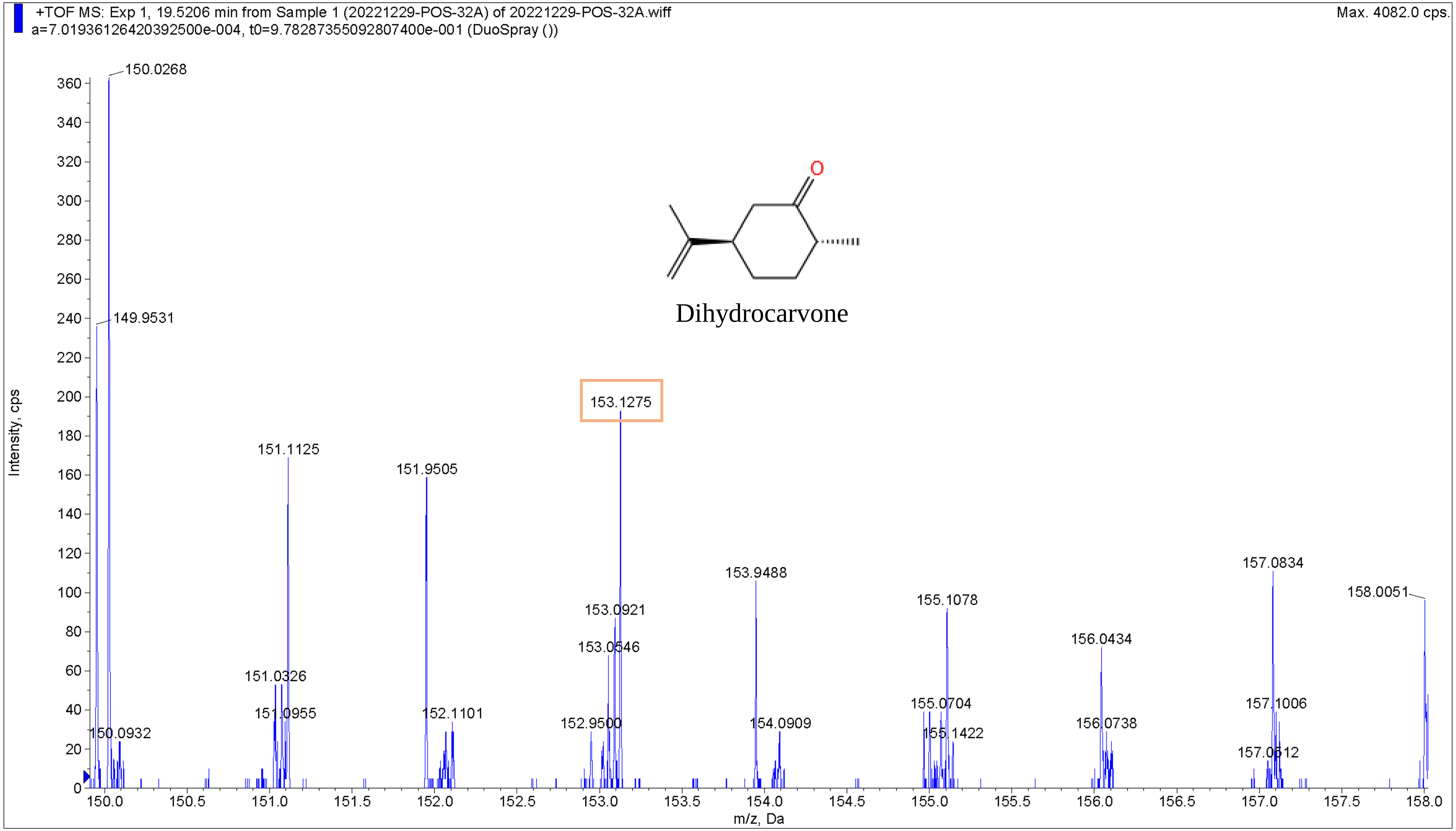
+TOF MS: Exp 1, 19.5206 min from Sample 1 (20221229-POS-32A) of 20221229-POS-32A.wiff
a=7.01936126420392500e-004, t0=9.78287355092807400e-001 (DuoSpray ())
Max. 4082.0 cps.
Dihydrocarvone
O
Intensity, cps
m/z, Da
150.0268
149.9531
151.1125
151.9505
153.1275
153.0921
153.0546
153.9488
155.1078
156.0434
157.0834
158.0051
151.0326
151.0955
152.1101
150.0932
152.9500
154.0909
155.0704
155.1422
156.0738
157.1006
157.0612

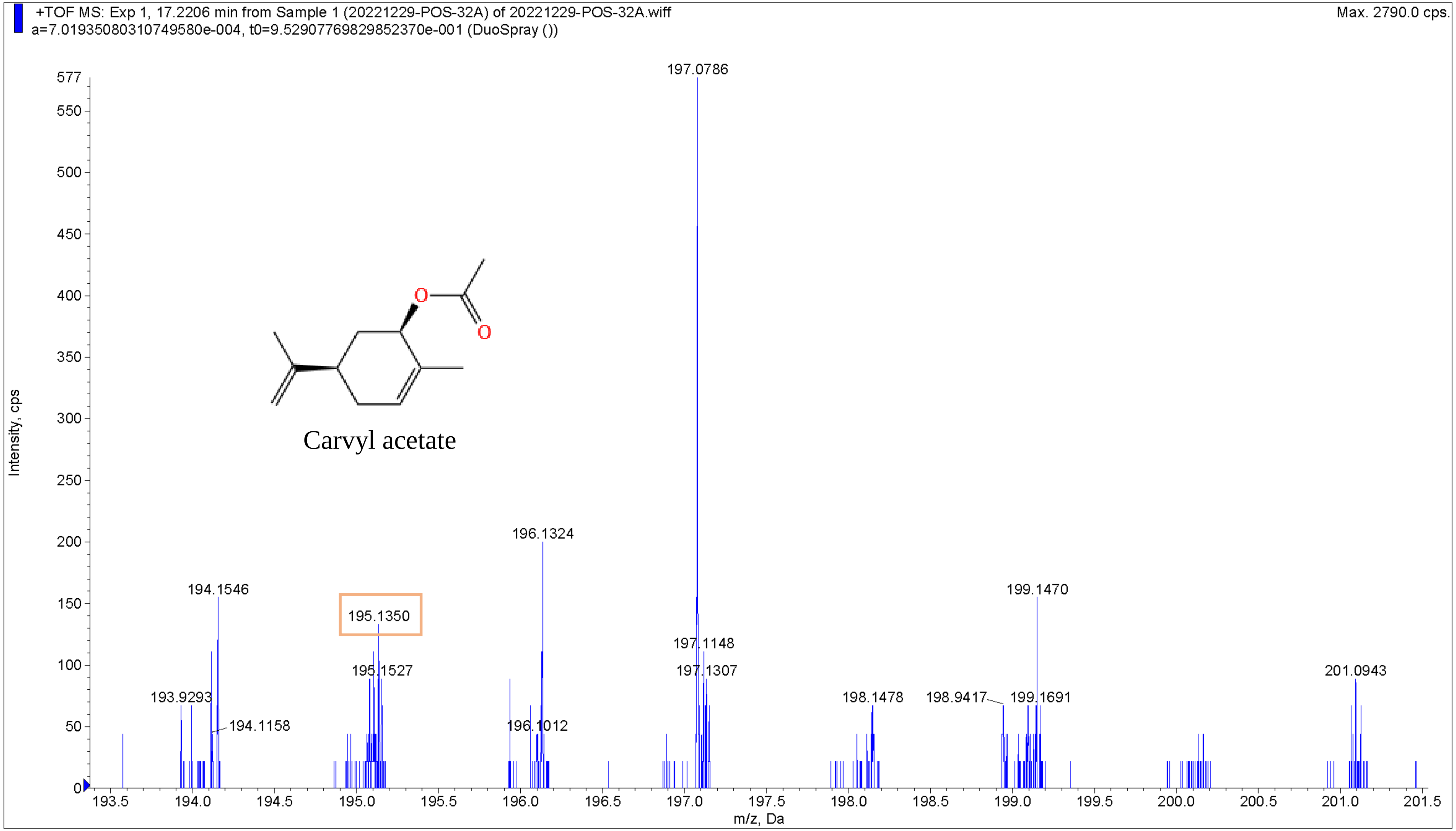
+TOF MS: Exp 1, 17.2206 min from Sample 1 (20221229-POS-32A) of 20221229-POS-32A.wiff
a=7.01935080310749580e-004, t0=9.52907769829852370e-001 (DuoSpray ())
Max. 2790.0 cps.
Carvyl acetate
197.0786
196.1324
194.1546
195.1350
199.1470
197.1148
197.1307
195.1527
201.0943
193.9293
198.1478
198.9417
199.1691
194.1158
196.1012
Intensity, cps
m/z, Da

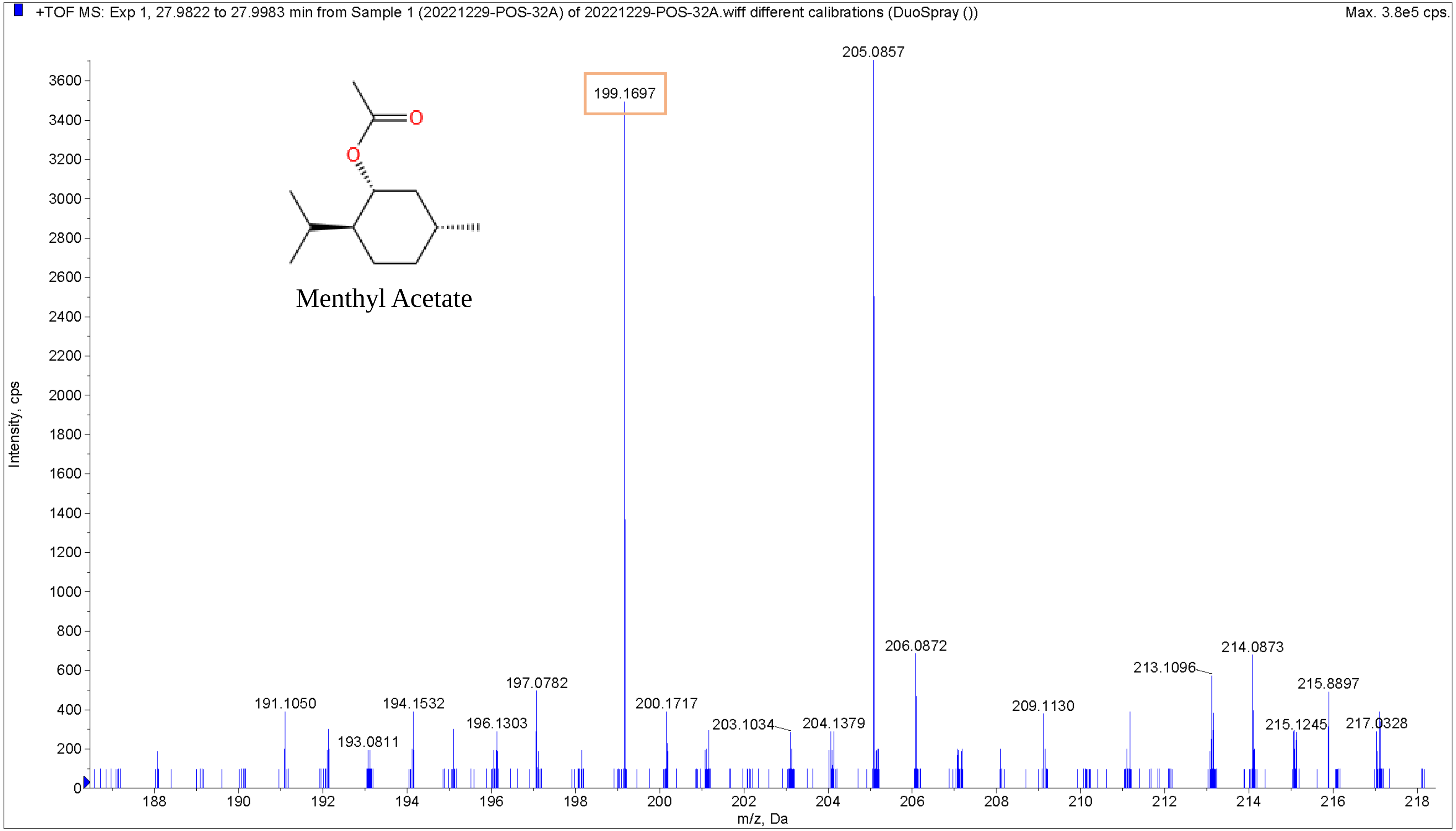

+TOF MS: Exp 1, 27.9822 to 27.9983 min from Sample 1 (20221229-POS-32A) of 20221229-POS-32A.wiff different calibrations (DuoSpray ())
Max. 3.8e5 cps.
Menthyl Acetate
199.1697
205.0857
Intensity, cps
m/z, Da
191.1050
193.0811
194.1532
196.1303
197.0782
200.1717
203.1034
204.1379
206.0872
209.1130
213.1096
214.0873
215.1245
215.8897
217.0328

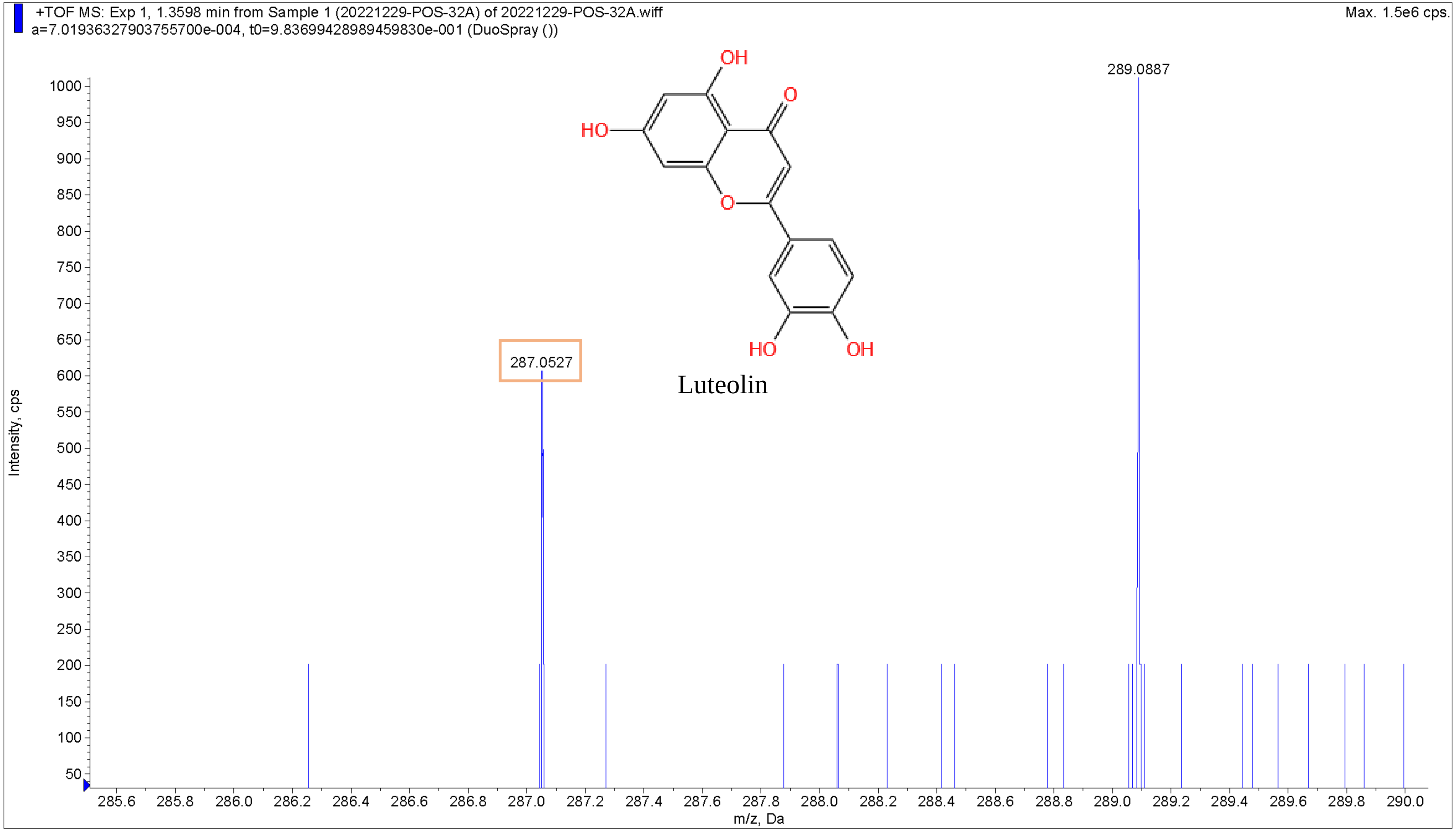

+TOF MS: Exp 1, 1.3598 min from Sample 1 (20221229-POS-32A) of 20221229-POS-32A.wiff
a=7.01936327903755700e-004, t0=9.83699428989459830e-001 (DuoSpray ())
Max. 1.5e6 cps.
Intensity, cps
m/z, Da
289.0887
287.0527
Luteolin

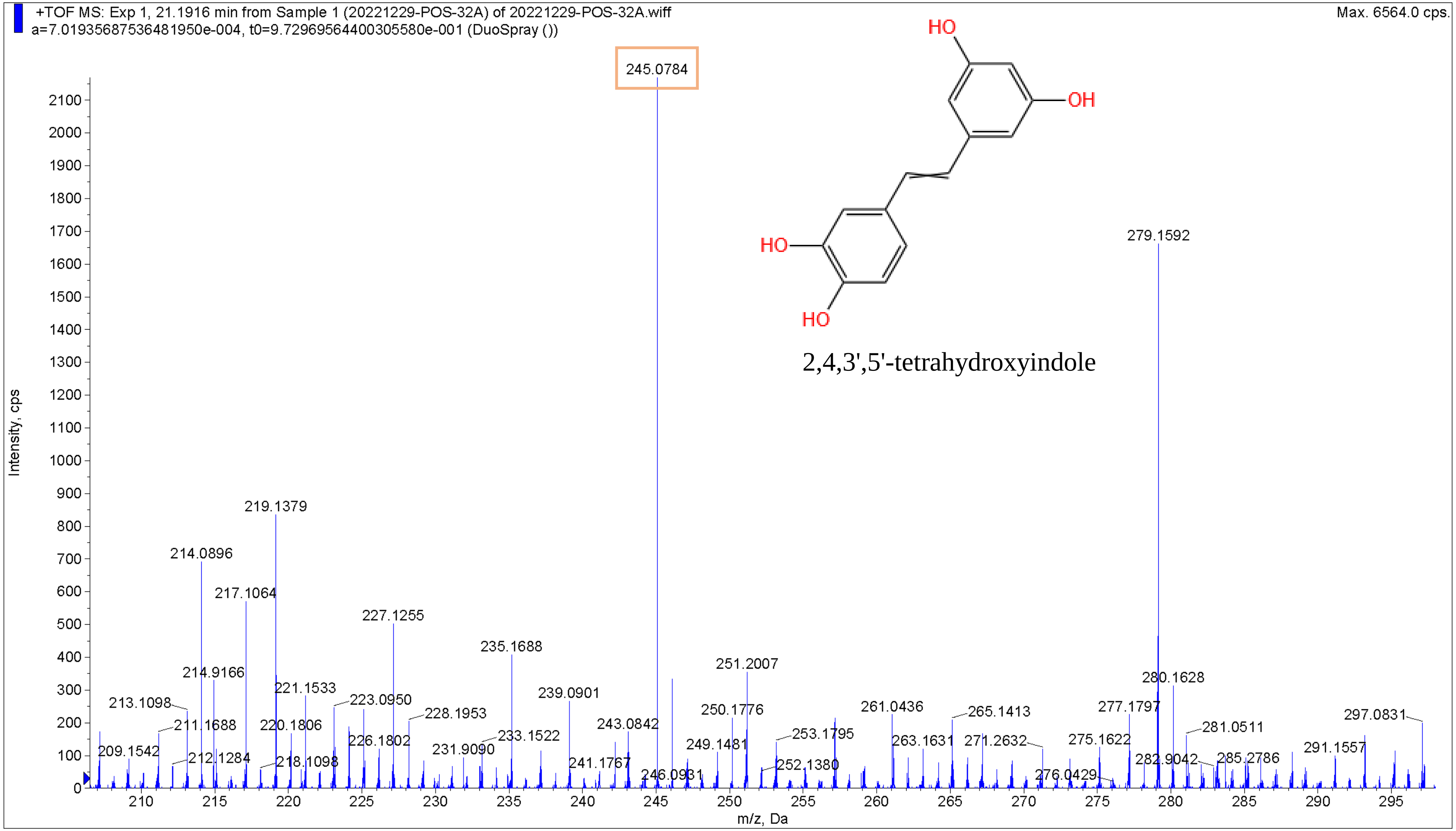
+TOF MS: Exp 1, 21.1916 min from Sample 1 (20221229-POS-32A) of 20221229-POS-32A.wiff
a=7.01935687536481950e-004, t0=9.72969564400305580e-001 (DuoSpray ())
Max. 6564.0 cps.
245.0784
279.1592
219.1379
214.0896
217.1064
227.1255
235.1688
251.2007
214.9166
221.1533
223.0950
228.1953
239.0901
250.1776
261.0436
265.1413
277.1797
280.1628
297.0831
213.1098
211.1688
220.1806
243.0842
253.1795
281.0511
209.1542
212.1284
218.1098
226.1802
231.9090
233.1522
241.1767
246.0931
249.1481
252.1380
263.1631
271.2632
275.1622
276.0429
282.9042
285.2786
291.1557
Intensity, cps
m/z, Da
HO
OH
HO
HO
2,4,3',5'-tetrahydroxyindole

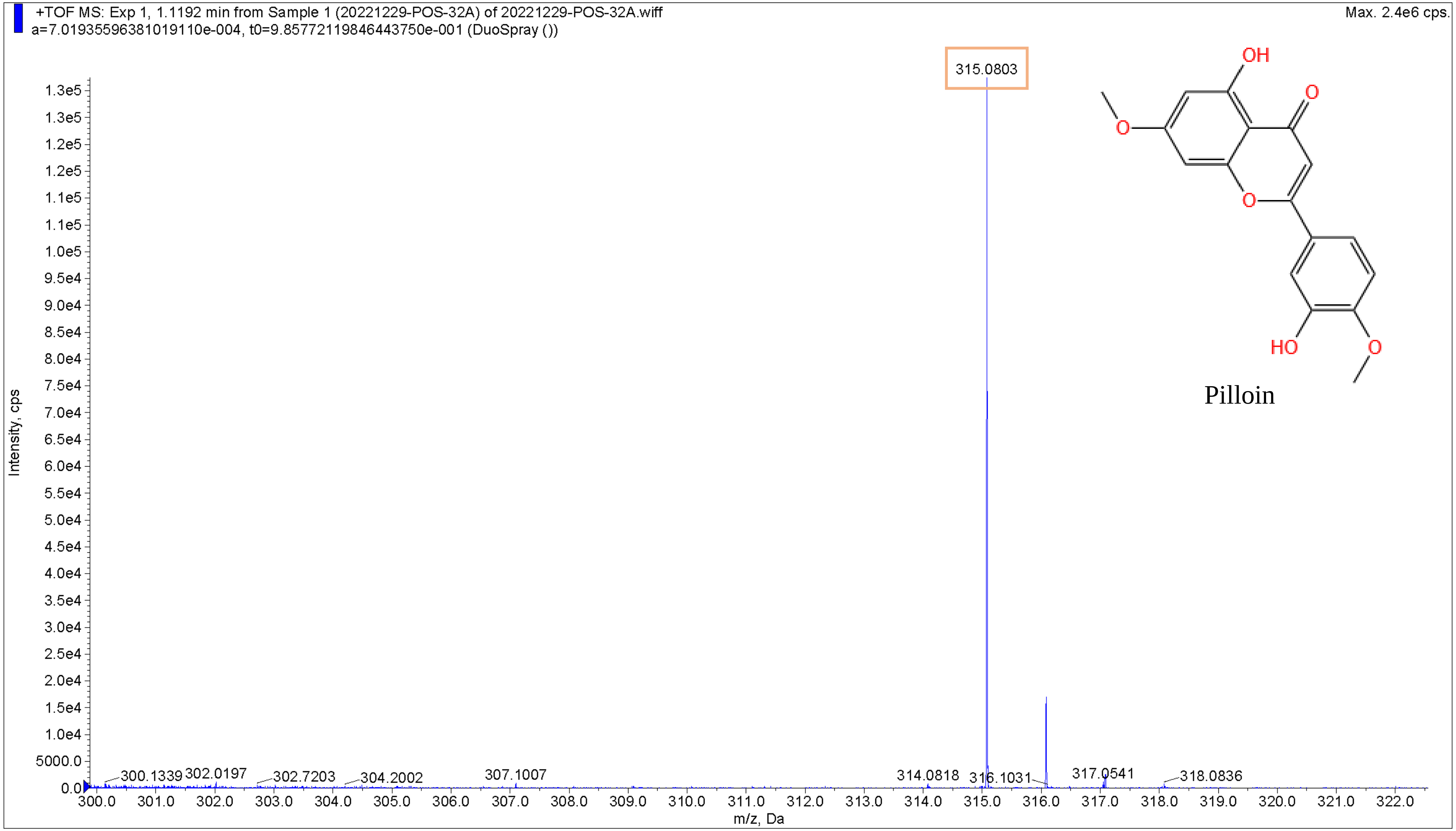

+TOF MS: Exp 1, 1.1192 min from Sample 1 (20221229-POS-32A) of 20221229-POS-32A.wiff
a=7.01935596381019110e-004, t0=9.85772119846443750e-001 (DuoSpray ())
Max. 2.4e6 cps.
315.0803
Pilloin
Intensity, cps
m/z, Da
300.1339
302.0197
302.7203
304.2002
307.1007
314.0818
316.1031
317.0541
318.0836

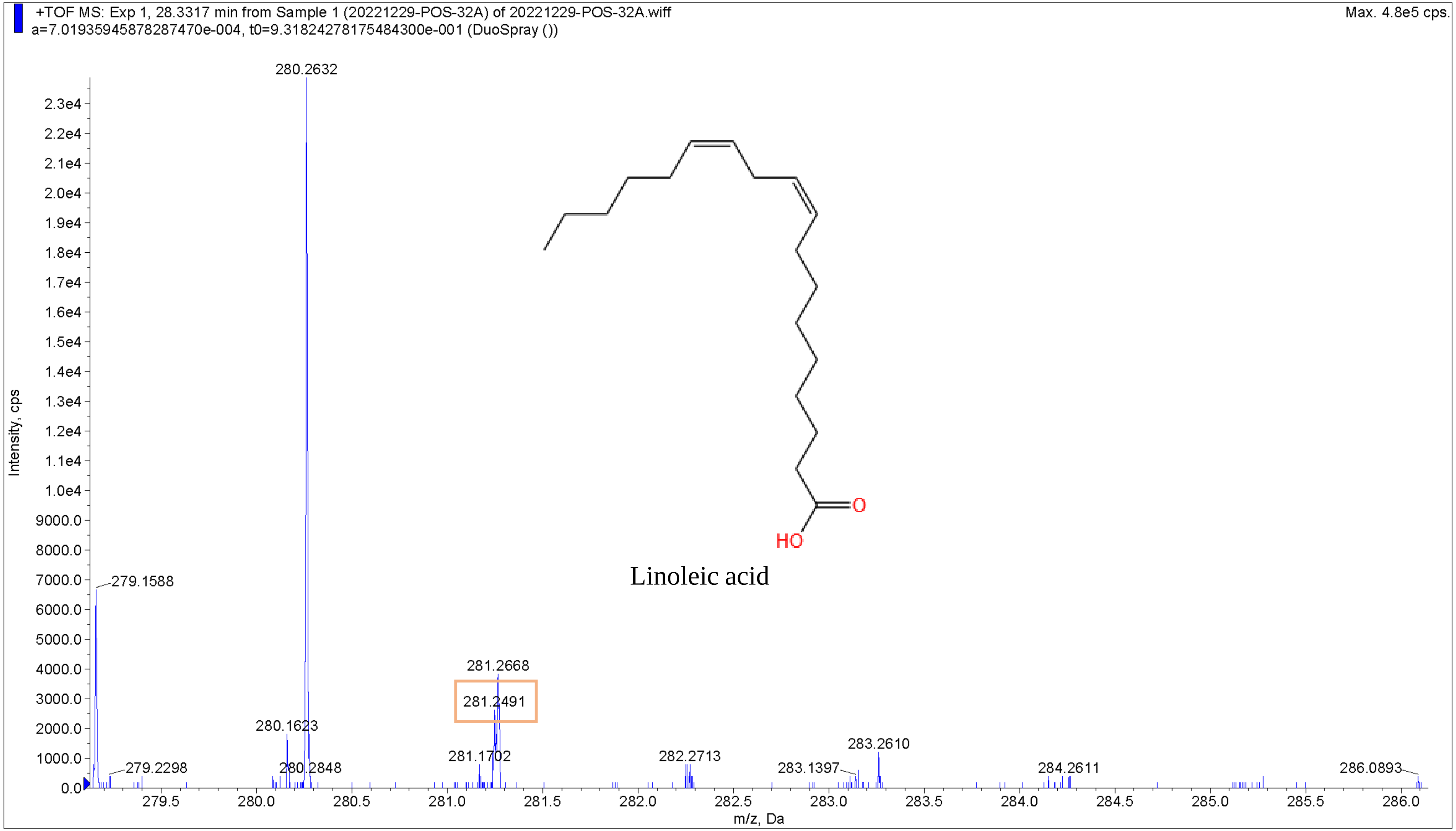

+TOF MS: Exp 1, 28.3317 min from Sample 1 (20221229-POS-32A) of 20221229-POS-32A.wiff
a=7.01935945878287470e-004, t0=9.31824278175484300e-001 (DuoSpray ())
Max. 4.8e5 cps.
280.2632
279.1588
279.2298
280.1623
280.2848
281.2668
281.2491
281.1702
282.2713
283.1397
283.2610
284.2611
286.0893
Intensity, cps
m/z, Da
HO
O
Linoleic acid

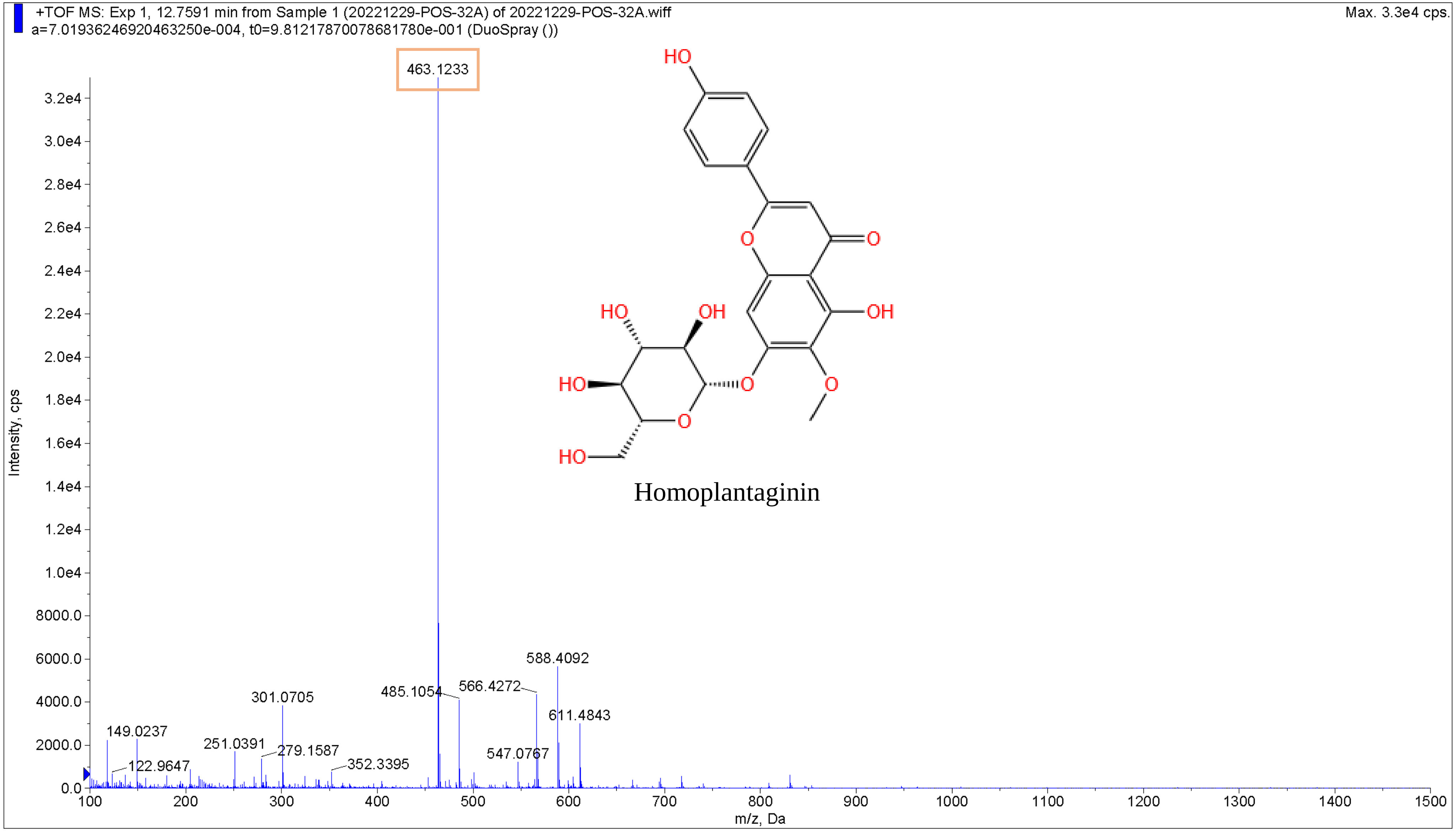
+TOF MS: Exp 1, 12.7591 min from Sample 1 (20221229-POS-32A) of 20221229-POS-32A.wiff
a=7.01936246920463250e-004, t0=9.81217870078681780e-001 (DuoSpray ())
Max. 3.3e4 cps.
463.1233
588.4092
485.1054
566.4272
611.4843
301.0705
149.0237
251.0391
279.1587
122.9647
352.3395
547.0767
Intensity, cps
m/z, Da
Homoplantaginin

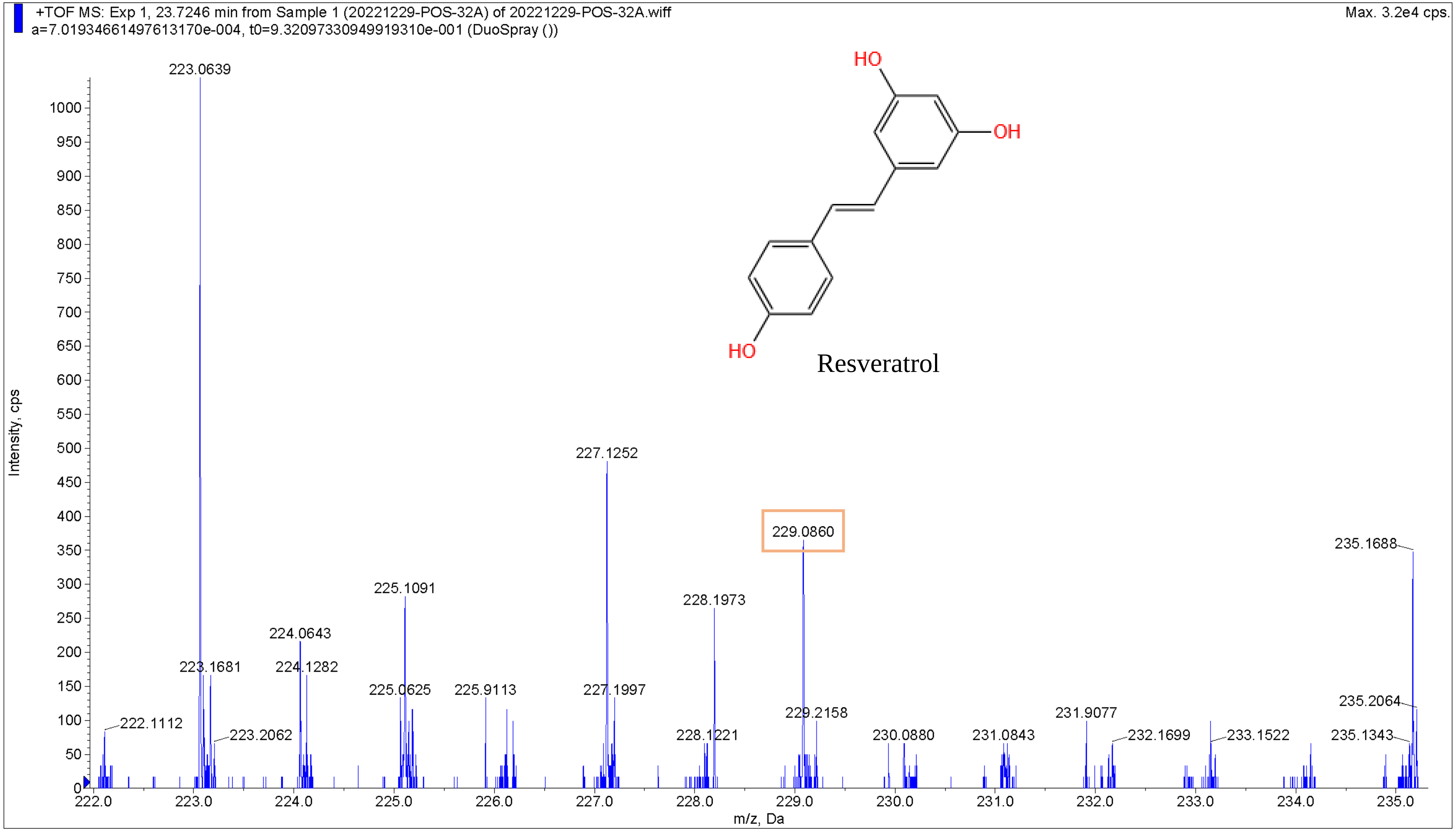
+TOF MS: Exp 1, 23.7246 min from Sample 1 (20221229-POS-32A) of 20221229-POS-32A.wiff
a=7.01934661497613170e-004, t0=9.32097330949919310e-001 (DuoSpray ())
Max. 3.2e4 cps.
Intensity, cps
m/z, Da
223.0639
227.1252
229.0860
235.1688
225.1091
228.1973
224.0643
223.1681
224.1282
225.0625
225.9113
227.1997
229.2158
235.2064
222.1112
223.2062
228.1221
230.0880
231.0843
231.9077
232.1699
233.1522
235.1343
HO
OH
HO
Resveratrol

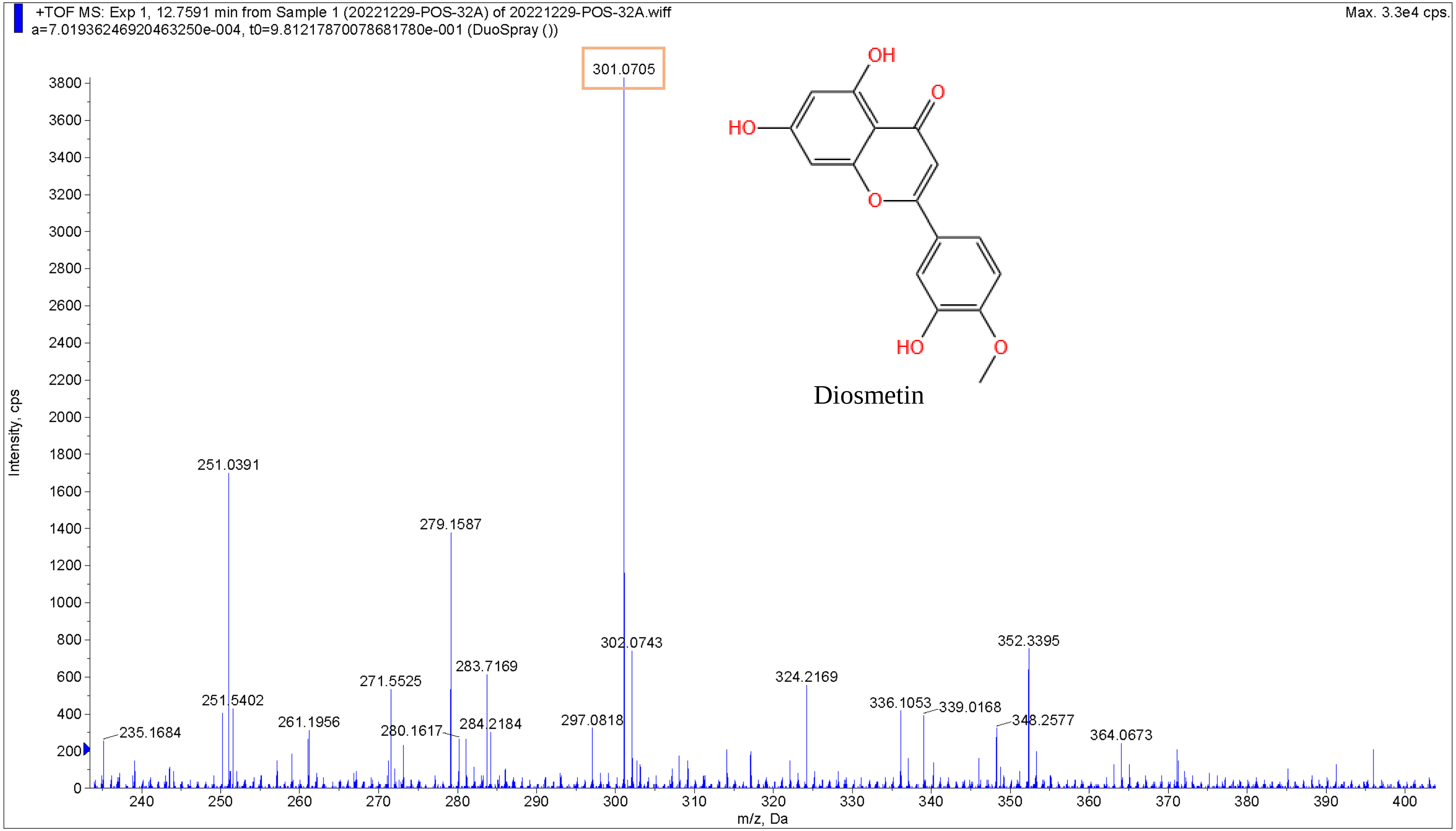
+TOF MS: Exp 1, 12.7591 min from Sample 1 (20221229-POS-32A) of 20221229-POS-32A.wiff
a=7.01936246920463250e-004, t0=9.81217870078681780e-001 (DuoSpray ())
Max. 3.3e4 cps.
301.0705
251.0391
279.1587
302.0743
283.7169
271.5525
251.5402
261.1956
235.1684
280.1617
284.2184
297.0818
324.2169
336.1053
339.0168
348.2577
352.3395
364.0673
Intensity, cps
m/z, Da
Diosmetin

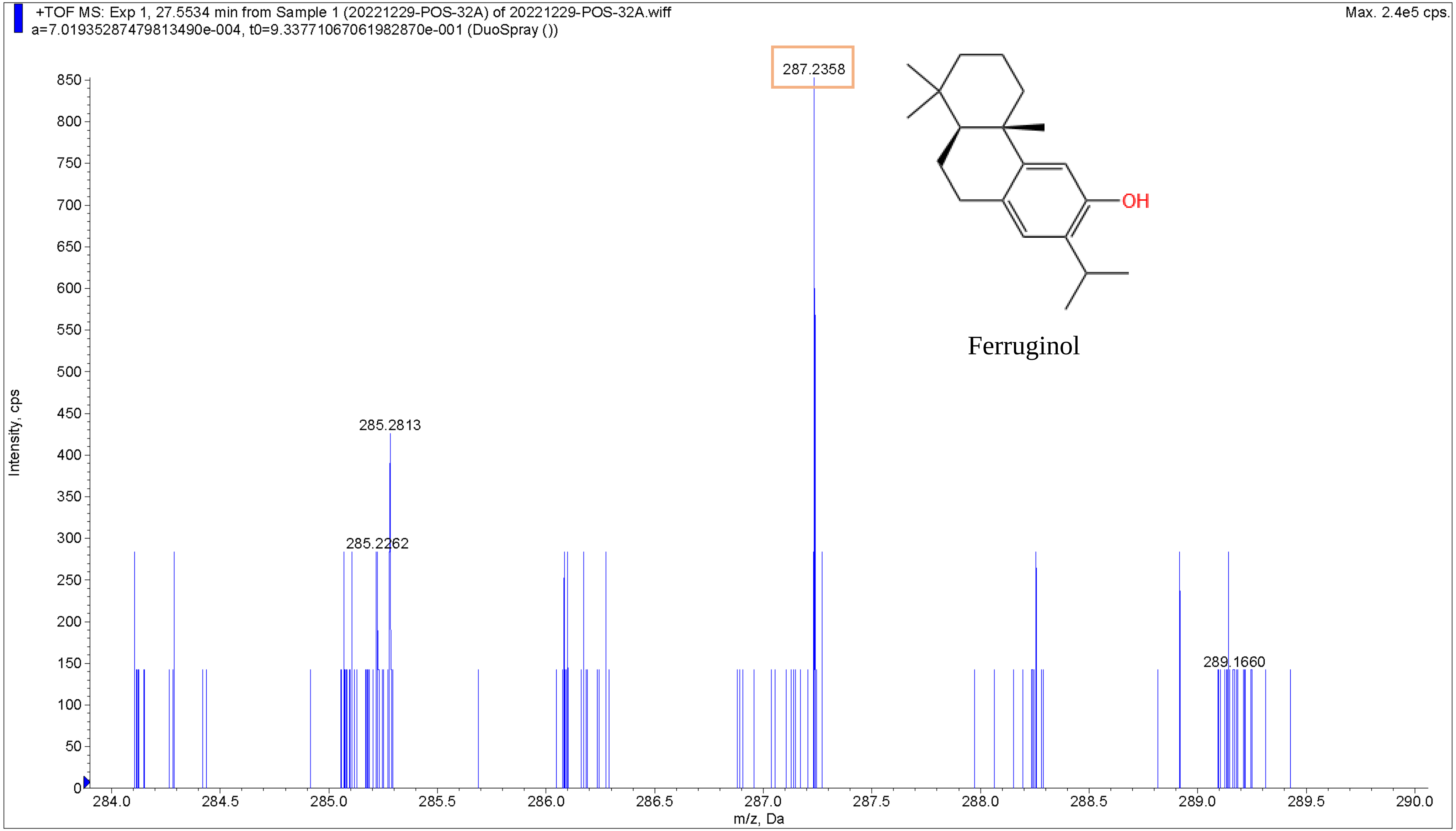

+TOF MS: Exp 1, 27.5534 min from Sample 1 (20221229-POS-32A) of 20221229-POS-32A.wiff
a=7.01935287479813490e-004, t0=9.33771067061982870e-001 (DuoSpray ())
Max. 2.4e5 cps.
287.2358
OH
Ferruginol
285.2813
285.2262
289.1660
Intensity, cps
m/z, Da

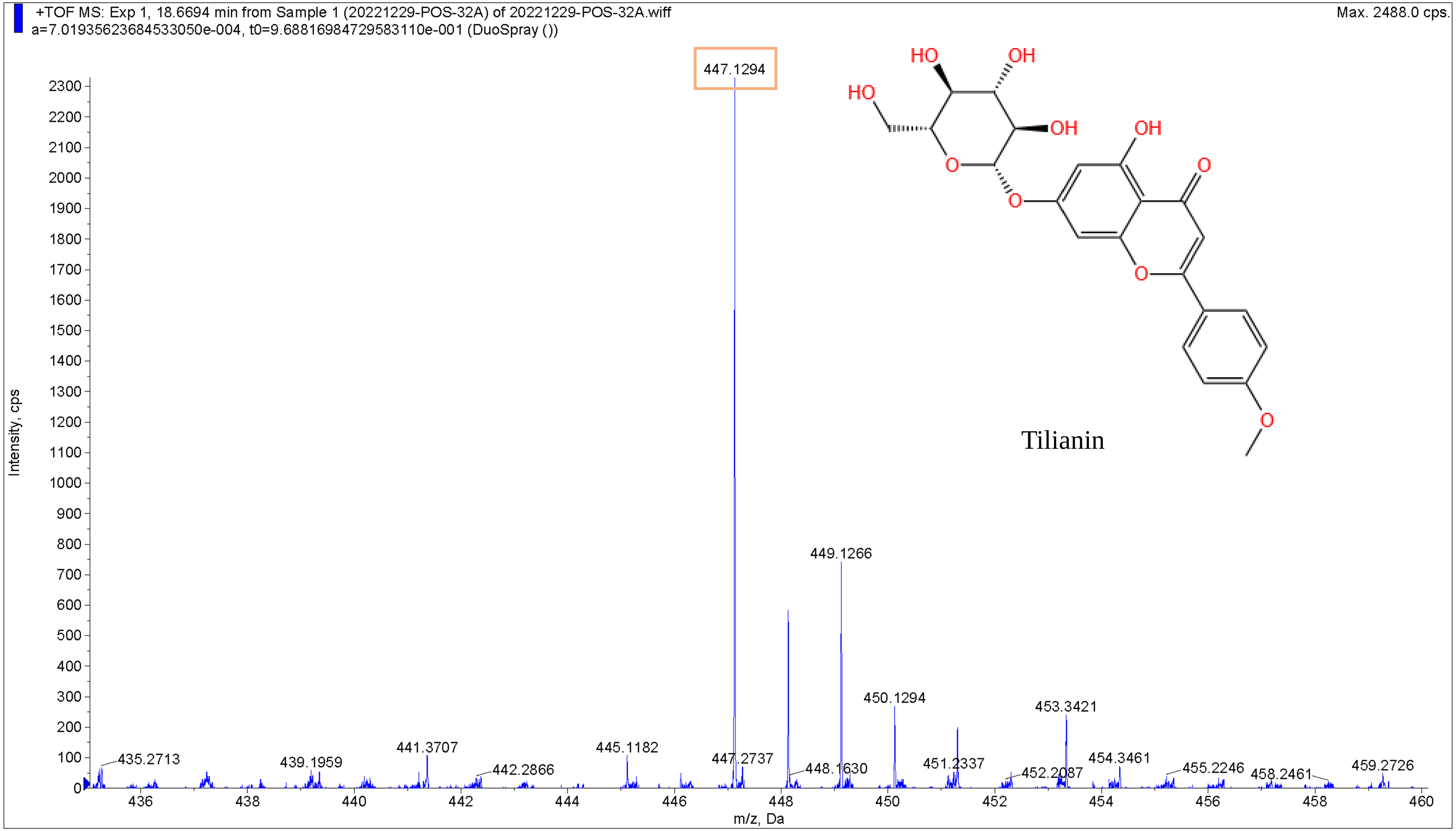

+TOF MS: Exp 1, 18.6694 min from Sample 1 (20221229-POS-32A) of 20221229-POS-32A.wiff
a=7.01935623684533050e-004, t0=9.68816984729583110e-001 (DuoSpray ())
Max. 2488.0 cps.
447.1294
449.1266
450.1294
453.3421
435.2713
439.1959
441.3707
442.2866
445.1182
447.2737
448.1630
451.2337
452.2087
454.3461
455.2246
458.2461
459.2726
Intensity, cps
m/z, Da
HO
OH
HO
OH
OH
O
O
O
O
O
Tilianin

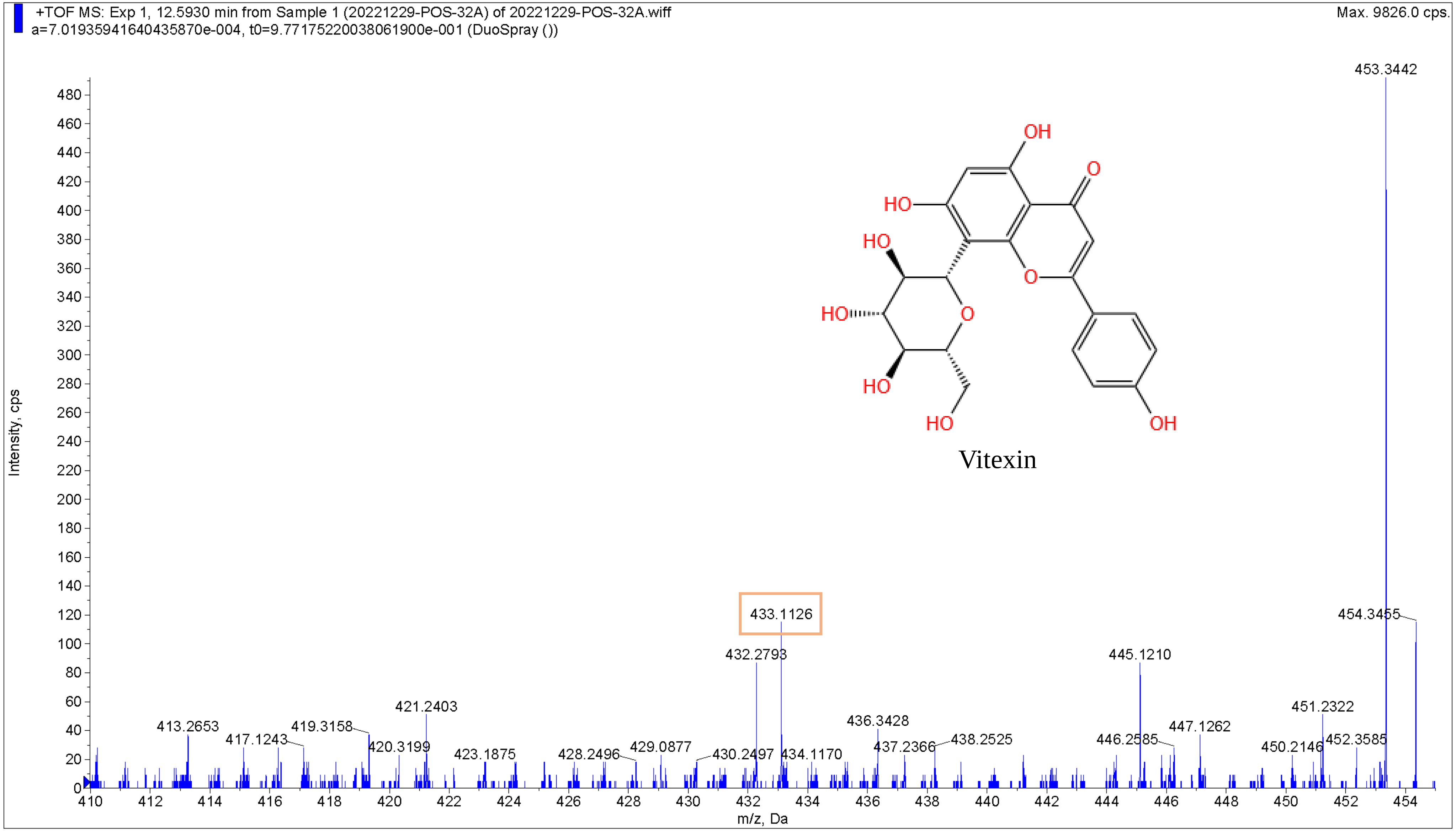
+TOF MS: Exp 1, 12.5930 min from Sample 1 (20221229-POS-32A) of 20221229-POS-32A.wiff
a=7.01935941640435870e-004, t0=9.77175220038061900e-001 (DuoSpray ())
Max. 9826.0 cps.
Intensity, cps
m/z, Da
453.3442
454.3455
433.1126
432.2793
445.1210
421.2403
451.2322
413.2653
419.3158
447.1262
436.3428
417.1243
420.3199
429.0877
446.2585
438.2525
452.3585
437.2366
450.2146
423.1875
428.2496
430.2497
434.1170
Vitexin

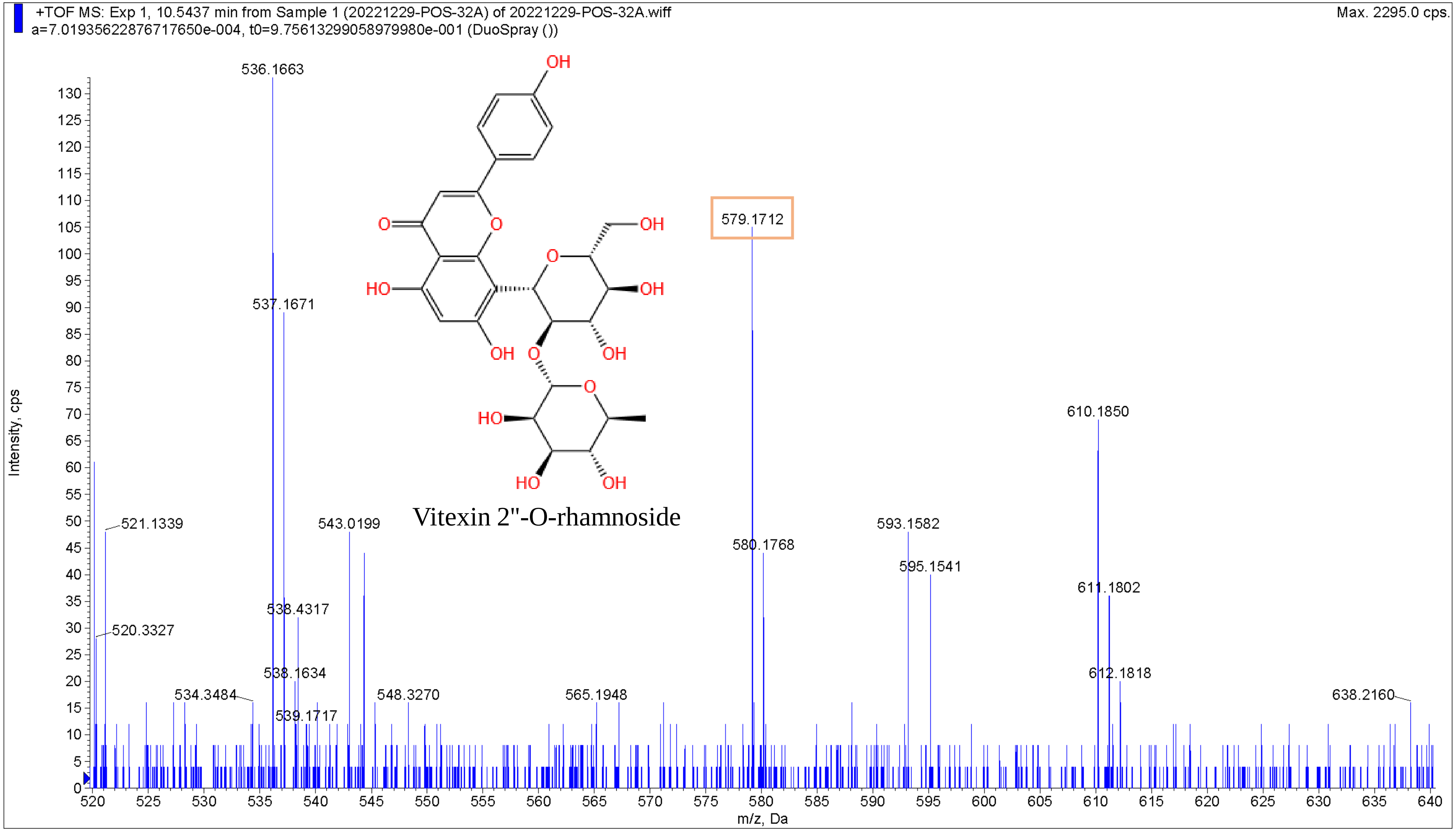
+TOF MS: Exp 1, 10.5437 min from Sample 1 (20221229-POS-32A) of 20221229-POS-32A.wiff
a=7.01935622876717650e-004, t0=9.75613299058979980e-001 (DuoSpray ())
Max. 2295.0 cps.
Intensity, cps
m/z, Da
536.1663
537.1671
538.4317
538.1634
539.1717
534.3484
521.1339
520.3327
543.0199
548.3270
565.1948
579.1712
580.1768
593.1582
595.1541
610.1850
611.1802
612.1818
638.2160
Vitexin 2''-O-rhamnoside

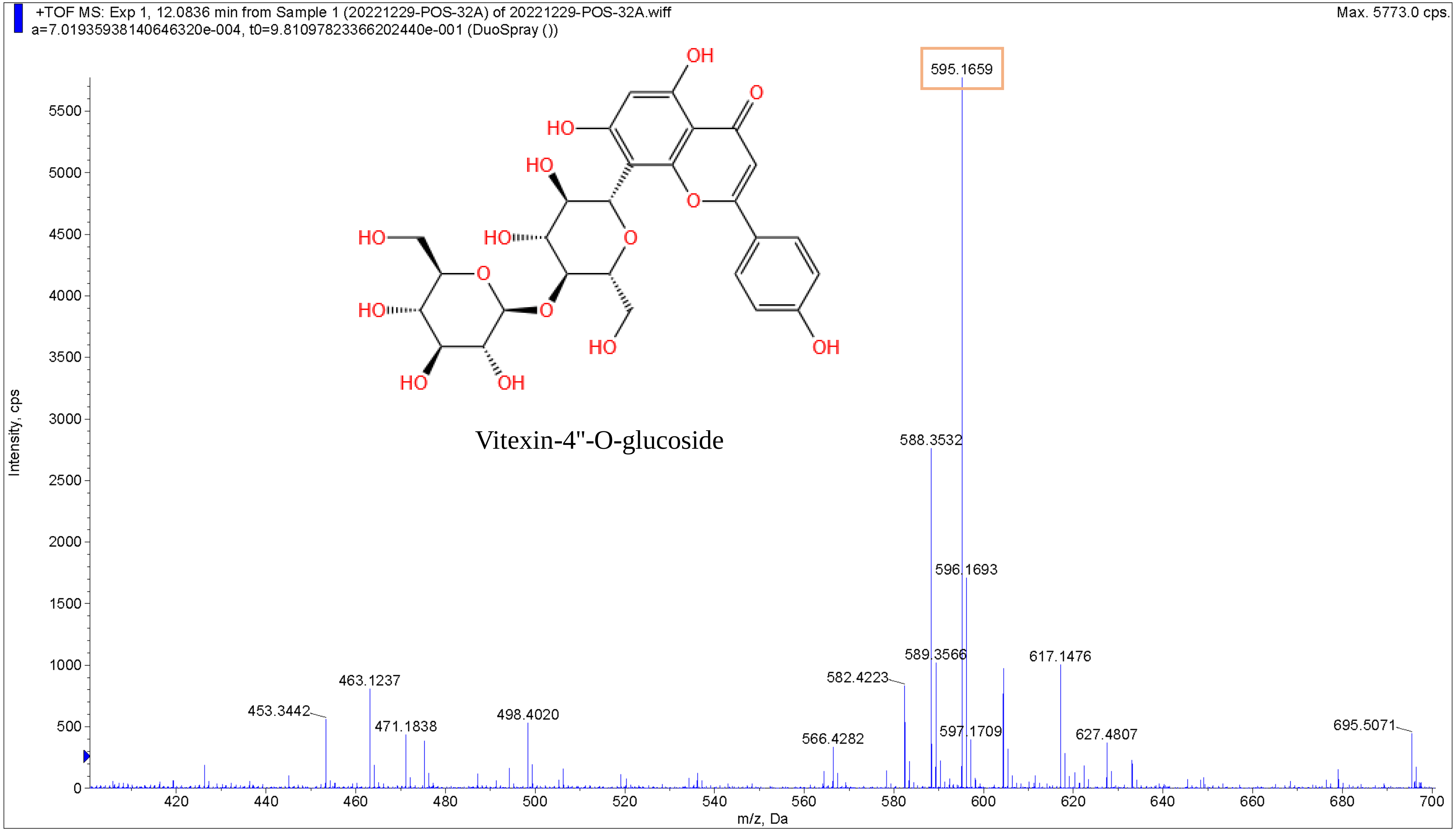
+TOF MS: Exp 1, 12.0836 min from Sample 1 (20221229-POS-32A) of 20221229-POS-32A.wiff
a=7.01935938140646320e-004, t0=9.81097823366202440e-001 (DuoSpray ())
Max. 5773.0 cps.
Vitexin-4"-O-glucoside
595.1659
588.3532
596.1693
589.3566
617.1476
582.4223
463.1237
453.3442
471.1838
498.4020
566.4282
597.1709
627.4807
695.5071
Intensity, cps
m/z, Da

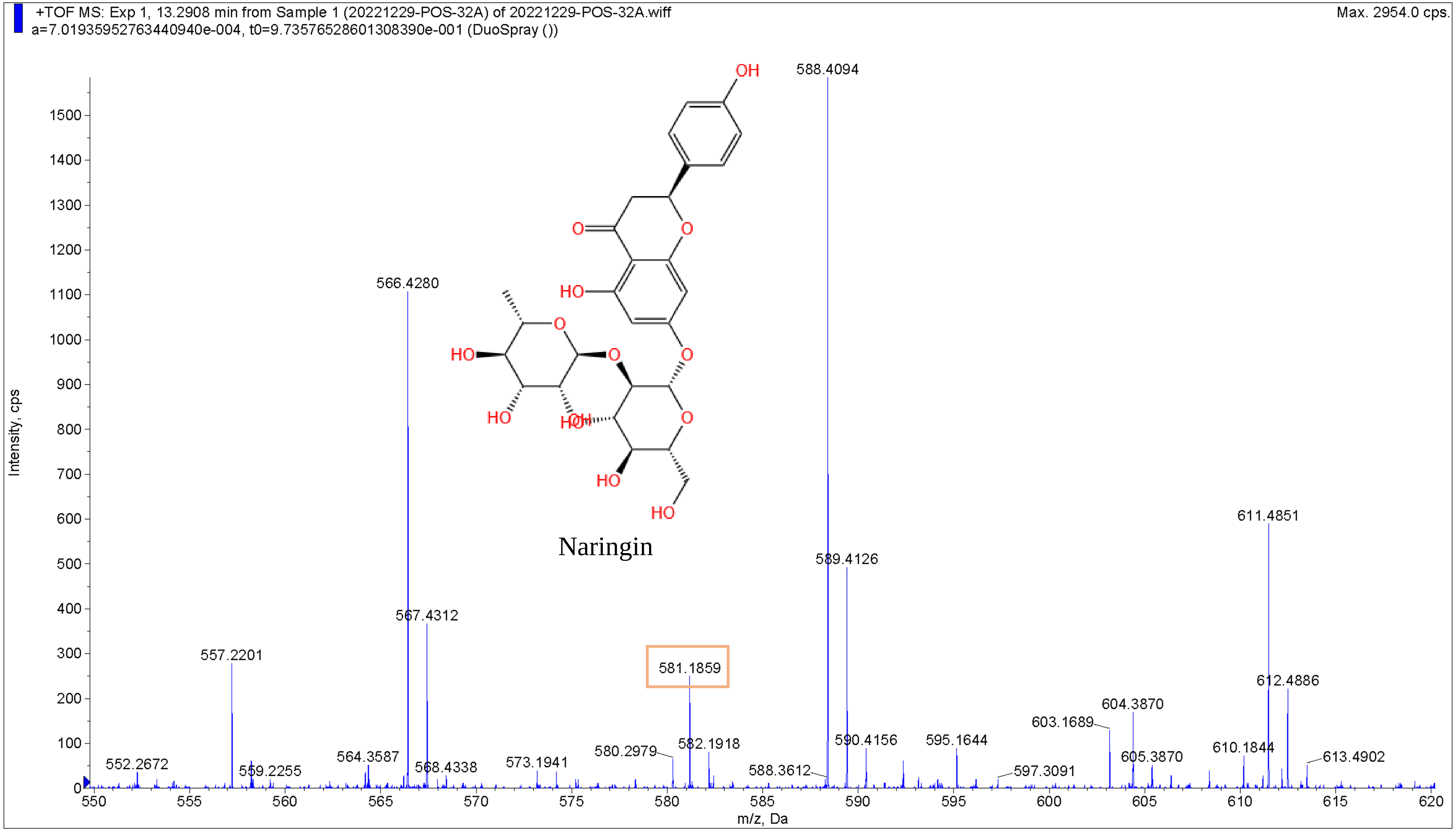

+TOF MS: Exp 1, 13.2908 min from Sample 1 (20221229-POS-32A) of 20221229-POS-32A.wiff
a=7.01935952763440940e-004, t0=9.73576528601308390e-001 (DuoSpray ())
Max. 2954.0 cps.
Intensity, cps
m/z, Da
588.4094
566.4280
611.4851
589.4126
567.4312
557.2201
581.1859
612.4886
604.3870
603.1689
582.1918
590.4156
595.1644
580.2979
552.2672
559.2255
564.3587
568.4338
573.1941
588.3612
597.3091
605.3870
610.1844
613.4902
Naringin

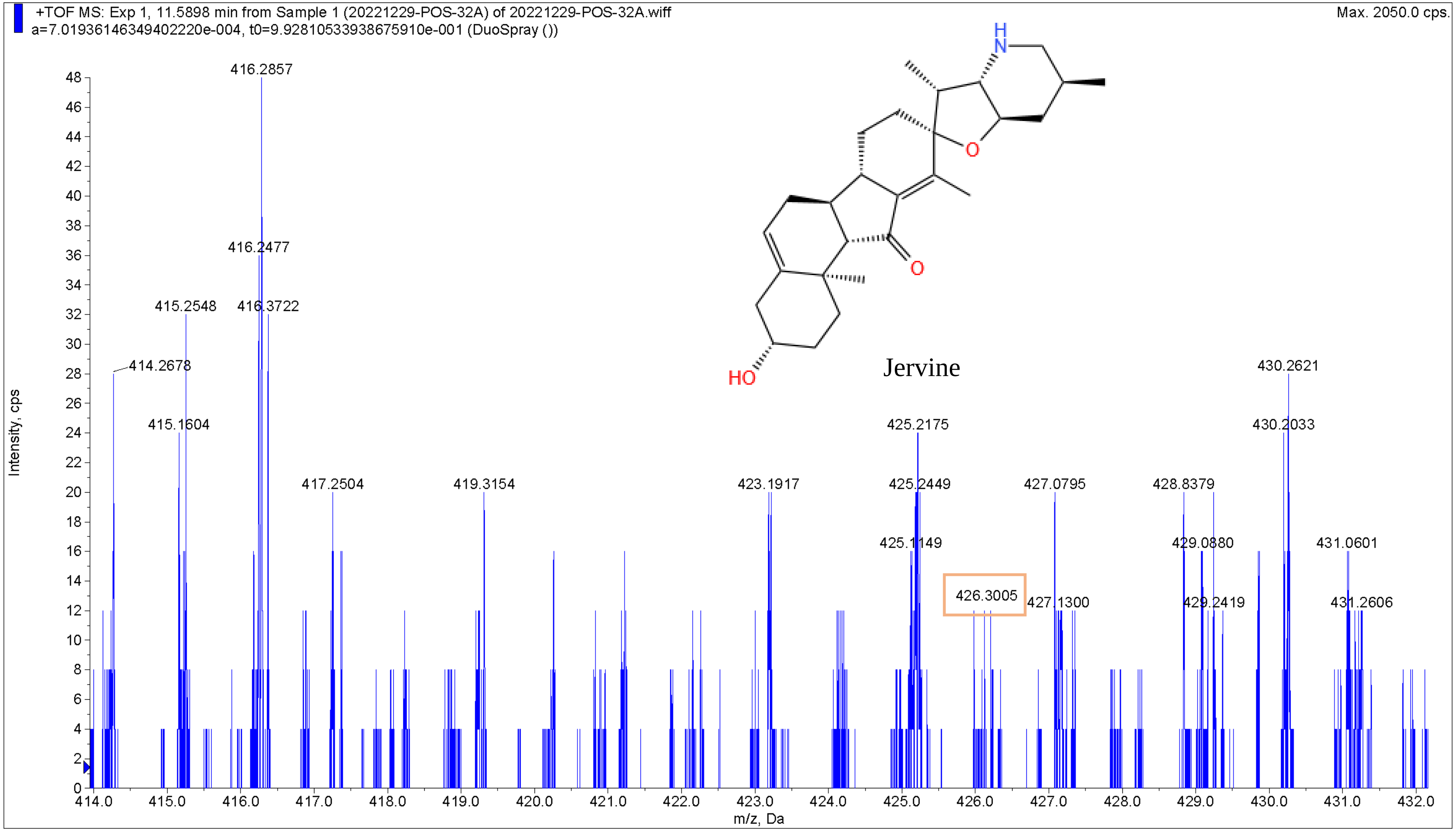

+TOF MS: Exp 1, 11.5898 min from Sample 1 (20221229-POS-32A) of 20221229-POS-32A.wiff
a=7.01936146349402220e-004, t0=9.92810533938675910e-001 (DuoSpray ())
Max. 2050.0 cps.
Intensity, cps
m/z, Da
414.2678
415.2548
415.1604
416.2857
416.2477
416.3722
417.2504
419.3154
423.1917
425.2175
425.2449
425.1149
426.3005
427.0795
427.1300
428.8379
429.0880
429.2419
430.2621
430.2033
431.0601
431.2606
Jervine

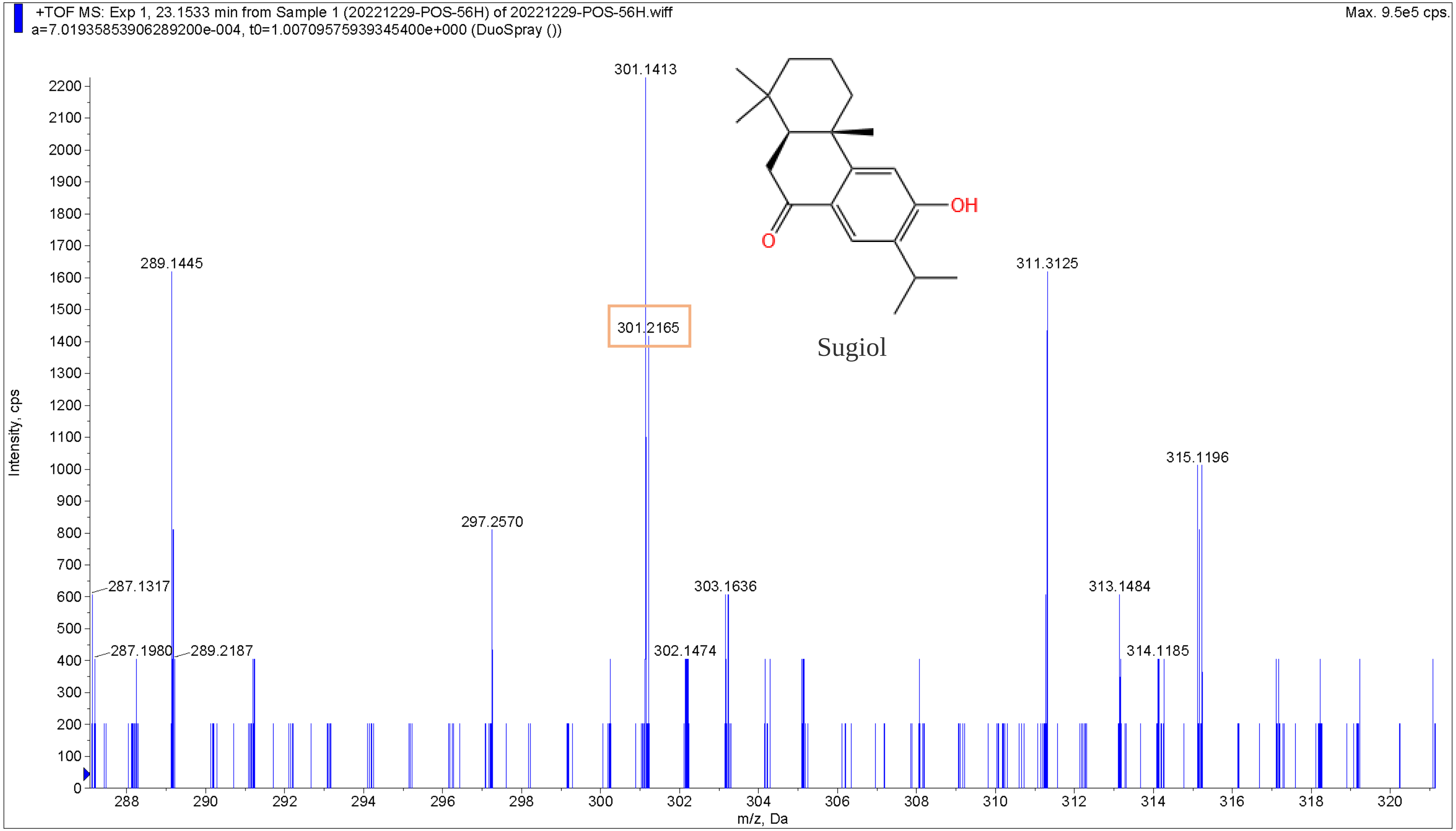

+TOF MS: Exp 1, 23.1533 min from Sample 1 (20221229-POS-56H) of 20221229-POS-56H.wiff
a=7.01935853906289200e-004, t0=1.00709575939345400e+000 (DuoSpray ())
Max. 9.5e5 cps.
Intensity, cps
m/z, Da
301.1413
301.2165
289.1445
311.3125
315.1196
297.2570
287.1317
303.1636
313.1484
287.1980
289.2187
302.1474
314.1185
OH
O
Sugiol

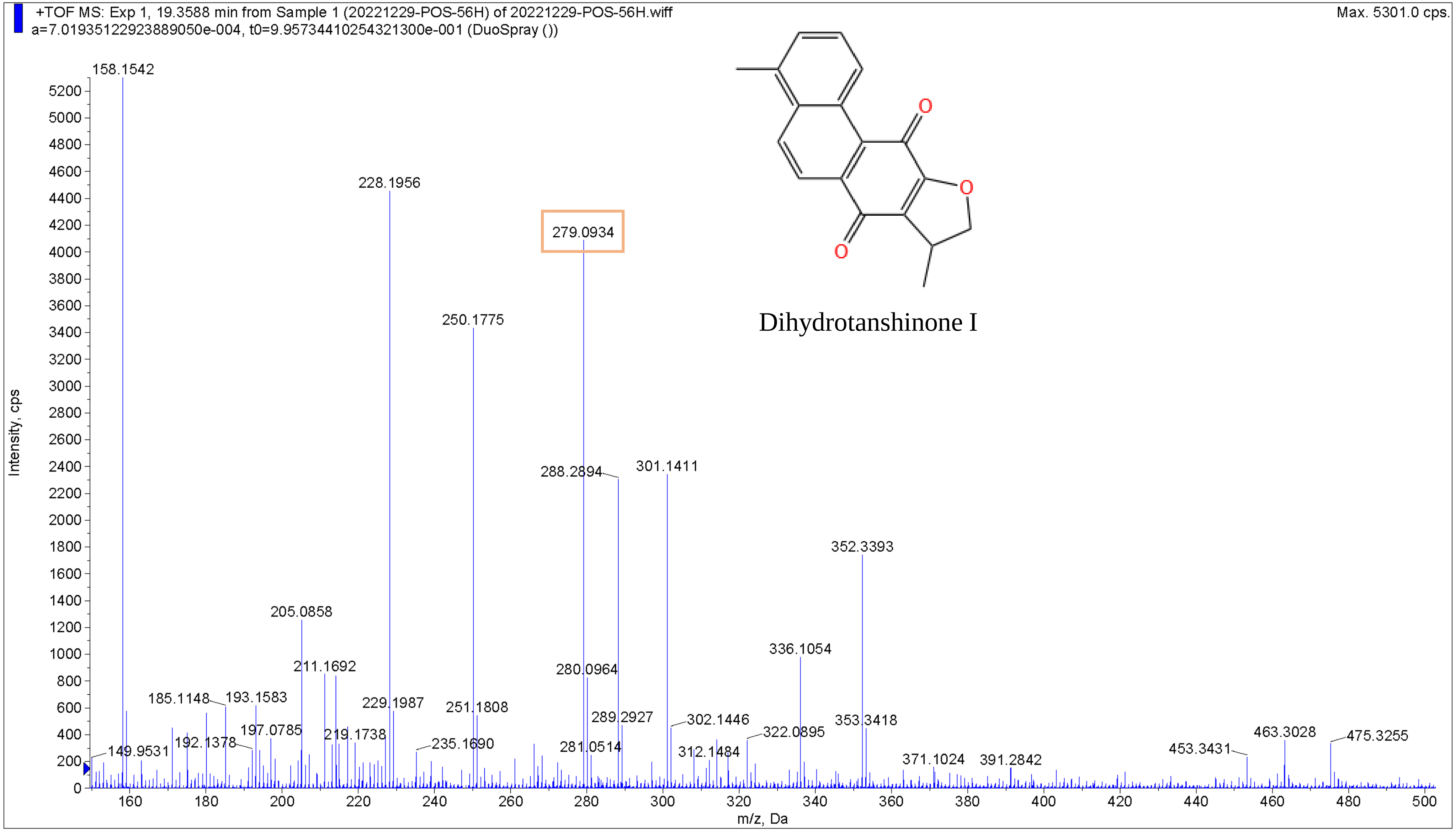
+TOF MS: Exp 1, 19.3588 min from Sample 1 (20221229-POS-56H) of 20221229-POS-56H.wiff
a=7.01935122923889050e-004, t0=9.95734410254321300e-001 (DuoSpray ())
Max. 5301.0 cps.
Intensity, cps
m/z, Da
158.1542
228.1956
279.0934
250.1775
288.2894
301.1411
352.3393
205.0858
336.1054
211.1692
280.0964
185.1148
193.1583
229.1987
251.1808
289.2927
302.1446
353.3418
197.0785
219.1738
322.0895
463.3028
475.3255
192.1378
235.1690
281.0514
312.1484
149.9531
453.3431
371.1024
391.2842
O
O
O
Dihydrotanshinone I

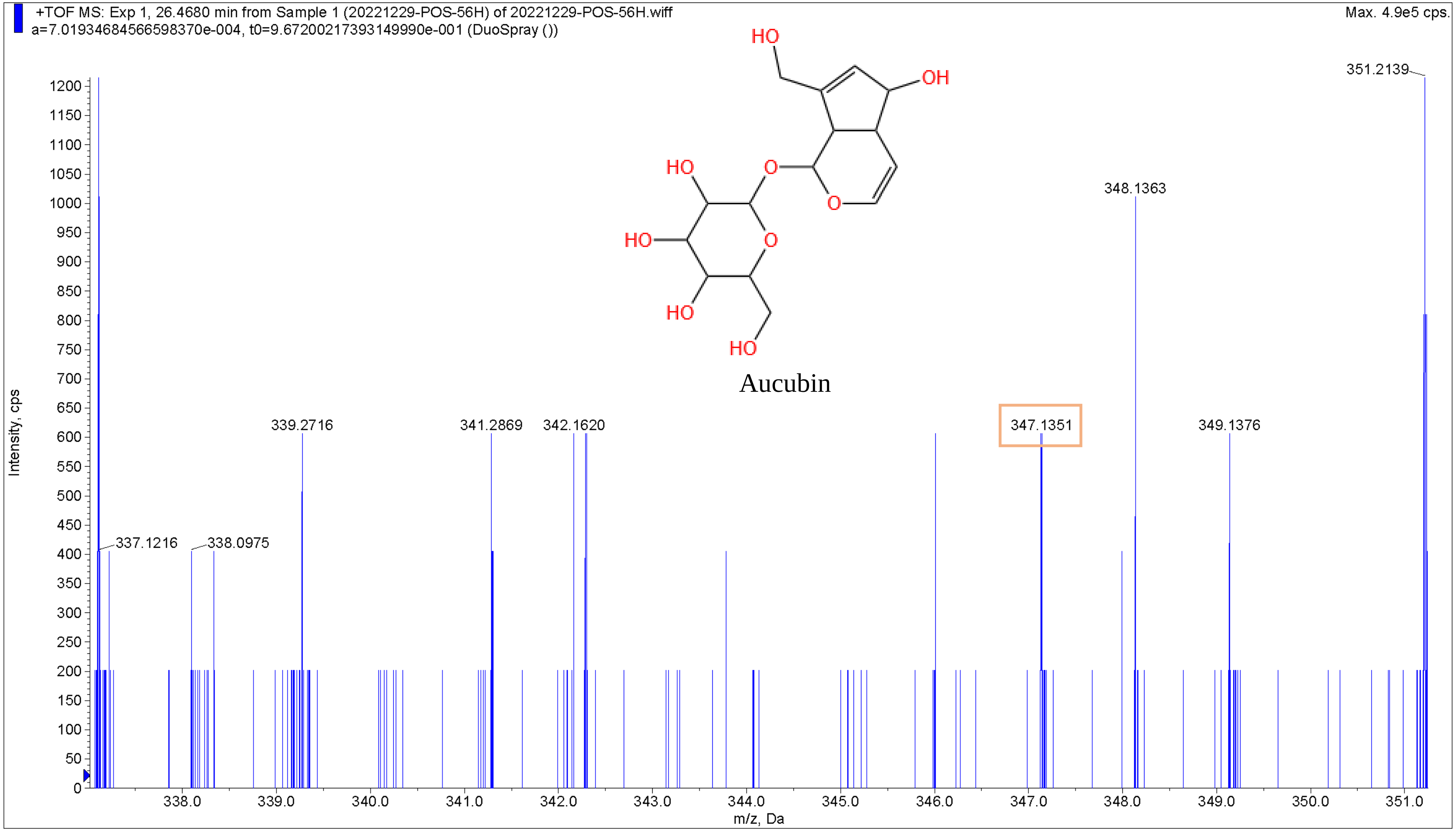
+TOF MS: Exp 1, 26.4680 min from Sample 1 (20221229-POS-56H) of 20221229-POS-56H.wiff
a=7.01934684566598370e-004, t0=9.67200217393149990e-001 (DuoSpray ())
Max. 4.9e5 cps.
Aucubin
HO
OH
O
351.2139
348.1363
347.1351
349.1376
339.2716
341.2869
342.1620
337.1216
338.0975
Intensity, cps
m/z, Da

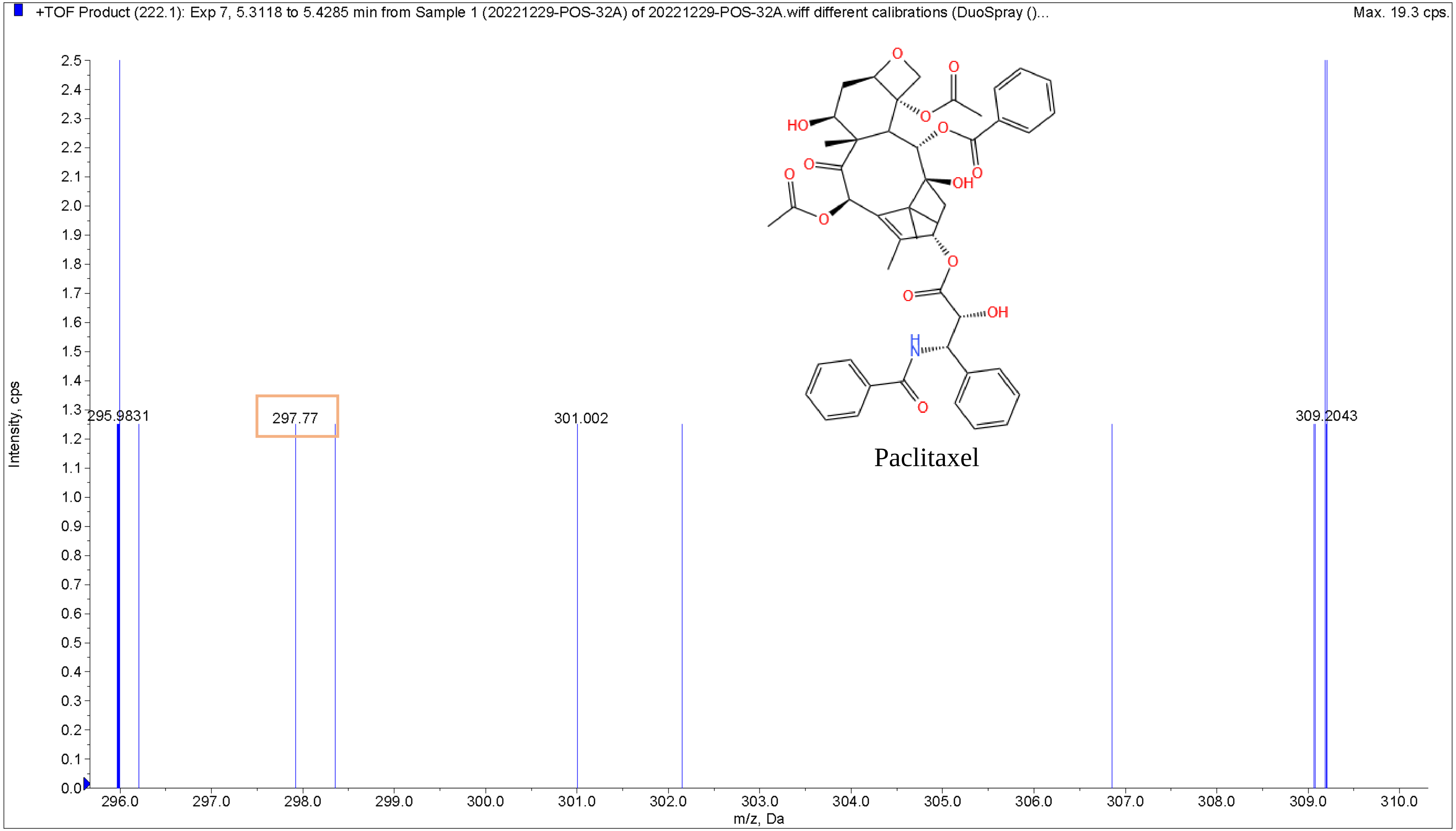

+TOF Product (222.1): Exp 7, 5.3118 to 5.4285 min from Sample 1 (20221229-POS-32A) of 20221229-POS-32A.wiff different calibrations (DuoSpray ()...
Max. 19.3 cps.
Intensity, cps
m/z, Da
295.9831
297.77
301.002
309.2043
HO
OH
O
H
N
Paclitaxel
